# Oral delivery of teriparatide utilizing biocompatible transferrin-engineered MOF nanoparticles for osteoporosis therapy

**DOI:** 10.1016/j.mtbio.2025.102318

**Published:** 2025-09-15

**Authors:** Renxiong Wei, Sang Hu, Jiazhi Wang, Qingjian Lei, Zhiyu Jiang, Bo Wang, Haixia Yang, Feifei Yan, Lin Cai, Jian Tian

**Affiliations:** a Department of Spine Surgery and Musculoskeletal Tumors, Zhongnan Hospital of Wuhan University, School of Pharmaceutical Sciences, Wuhan University, Wuhan, 430071, China; b State Key Laboratory of Metabolism and Regulation in Complex Organisms, College of Life Sciences, Wuhan University, Wuhan, 430071, China

**Keywords:** Osteoporosis, Metal-organic framework, Teriparatide, Oral delivery, Transferrin

## Abstract

Osteoporosis, a systemic skeletal disorder characterized by reduced bone density and increased fracture risk, poses a significant global health challenge. While teriparatide (TRP), a first-line anabolic peptide drug, demonstrates substantial therapeutic benefits in osteoporosis management, its clinical use is restricted by the necessity for daily subcutaneous administration, leading to suboptimal patient compliance. To overcome this limitation, we developed an orally deliverable TRP formulation using biocompatible metal-organic framework nanoparticles (MOF-808 NPs) co-loaded with TRP and functionalized with transferrin targeting ligands (M@P@T NPs). The rationally designed nanoporous architecture coupled with transferrin surface modification synergistically protects TRP from acidic and enzymatic degradation in harsh gastrointestinal environments, while realizing controlled release of TRP in the phosphate-rich bloodstream. Leveraging the overexpression of transferrin receptors (TfR) on intestinal epithelial cells, the nanosystem facilitates receptor-mediated transcellular transport, enabling efficient systemic delivery of TRP with high oral bioavailability. After the one-month oral administration of low-dose M@P@T (200 μg kg^−1^·day^−1^) to osteoporosis model mice, therapeutic outcomes comparable to those achieved with subcutaneous TRP injections were observed, including increased bone mineral density, improved trabecular structure, and significant alleviation of osteoporosis symptoms. These findings suggest that this MOF-based oral TRP strategy has great potential for simplifying and improving the treatment of osteoporosis.

## Highlights

• We develop an oral teriparatide (TRP) delivery system (M@P@T) that overcomes the need for daily subcutaneous injections.
• M@P@T protects TRP from digestion in the gastrointestinal environment and achieves controlled release in the bloodstream.
• Oral low-dose M@P@T shows comparable efficacy to subcutaneous TRP injections in bone repair and good biosafety *in vivo*.

## Graphical abstract

**Figure:**
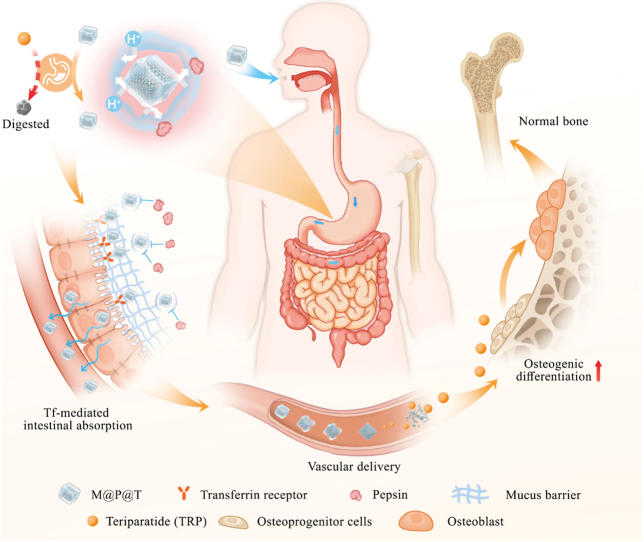

## 1 Introduction

Osteoporosis is a systemic bone metabolic disease characterized by decreased bone density, deterioration of the bone tissue microstructure, increased bone fragility, and a heightened risk of fractures [[1], [2], [3]]. Approximately 200 million individuals around the world suffer from osteoporosis, with approximately 8.9 million incidences of osteoporosis-related fractures reported annually, placing a significant burden on global healthcare resources and financial expenditures [4]. Current therapeutic drugs for osteoporosis can be divided into two categories: those that inhibit bone resorption and those that stimulate bone formation [[5], [6], [7]]. Bisphosphonates are the most commonly used antiresorptive drugs, but their long-term oral consumption carries the risk of atypical femoral fractures [8,9]. There are two main categories of synthetic metabolic drugs currently available for stimulating bone formation [10]. Among them, teriparatide (TRP), a 1–34 amino acid fragment of recombinant human parathyroid hormone [PTH (1-34)], was the only anabolic agent approved by the Food and Drug Administration (FDA) for the treatment of osteoporosis [11,12]. TRP promotes bone formation by targeting the parathyroid hormone-1 receptor [13], which improves bone strength and significantly reduces the risk of both vertebral and nonvertebral fractures. However, TRP is administered through daily subcutaneous (SC) injection, which is highly inconvenient for patients due to delayed therapeutic effects and poor adherence to treatment plans required for chronic diseases. Therefore, there is an urgent need to develop an orally administered formulation of TRP that provides therapeutic effects comparable to those achieved through subcutaneous injection.

Currently, there are very few orally available protein-based drugs in clinical use. This limitation is primarily due to the significant physiological barriers present within the gastrointestinal tract (GI), such as gastric acid, proteases, the mucous layer, and the tight junctions of small intestinal epithelial cells [14]. Therefore, the main challenge of orally delivering protein drugs is to overcome the corrosive effects of the GI tract and achieve effective absorption through the small intestinal epithelial cells. Fortunately, researchers have developed various drug delivery systems to address this issue [[15], [16], [17], [18], [19]]. For instance, Subedi and co-workers reported a complex combination of L-lysine deoxycholic acid (LDA), deoxycholic acid (DA), and TRP that displayed improved intestinal membrane permeability and promoted absorption [20]. Moreover, Zou and co-workers showed that transferrin-coated acid-resistant metal-organic framework nanoparticles (MOF NPs) protected insulin and significantly increased its oral bioavailability to 29.6 % [21]. However, most literature reports provide only short-term data on therapeutic efficacy and biocompatibility, without addressing the potential long-term implications of oral administration. This aspect is crucial for managing chronic diseases such as osteoporosis. Therefore, it is essential to develop oral protein delivery systems that are easy to prepare, have high oral bioavailability, and ensure long-term biological safety.

In recent years, nanoscale metal–organic frameworks (nMOFs) have garnered widespread attention due to their unique advantages [[22], [23], [24]]. nMOFs are nanomaterials composed of metal ions and organic ligands and have numerous benefits as nanocarriers [[25], [26], [27]]. Firstly, nMOFs offer a large pore volume with tunable pore sizes [28], facilitating efficient drug loading while excluding large molecules, such as proteases, thereby effectively protecting the encapsulated protein drugs [[29], [30], [31]]. Secondly, nMOFs exhibit excellent stability and slowly degrade under specific conditions to release their encapsulated drugs [32]. Most importantly, as carriers for oral medications intended for the long-term treatment of chronic diseases, a variety of nMOFs exhibit good biocompatibility, ensuring easy clearance from the body after degradation without organ accumulation [[33], [34], [35]]. Hence, nMOFs have emerged as an ideal nanoplatform for oral drug delivery [36].

**Scheme 1:**
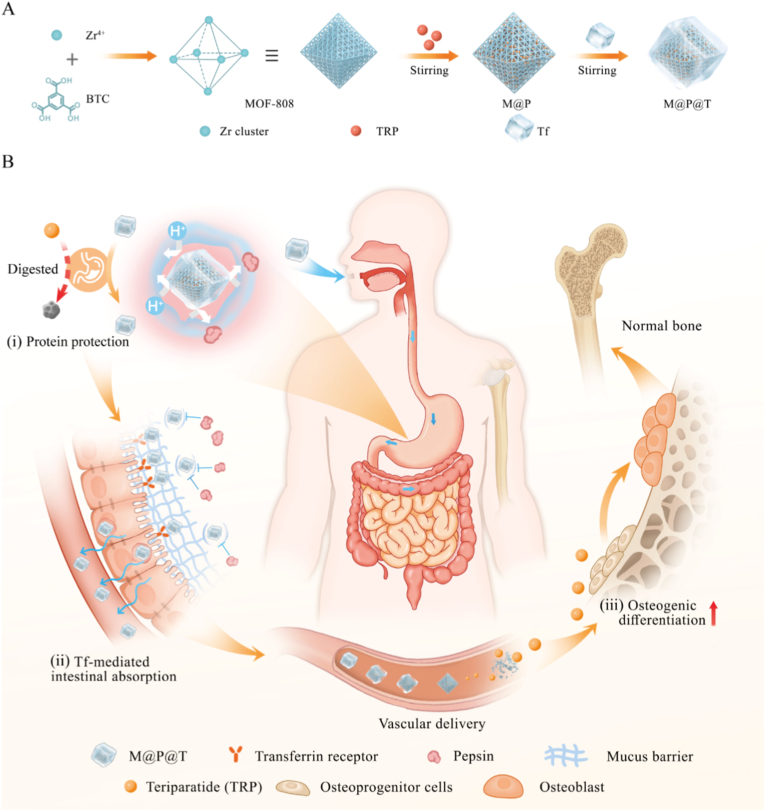
**Schematic illustration of the oral TRP-loaded MOF nanosystem for osteoporosis therapy.** (A) Schematic of the synthesis of the MOF-808-based nanosystems. (B) The M@P@T nanosystem not only protects TRP from gastric acid and enzymatic digestion (i) but also increases its specific absorption by intestinal epithelial cells (ii). The released TRP can promote osteogenic differentiation (iii), achieving effective osteoporosis treatment.

In this study, we fabricated a biocompatible oral peptide delivery nanosystem, M@P@T (M = MOF-808, P = TRP, T = Transferrin), for effective osteoporosis therapy (Scheme 1), utilizing acid-resistant Zr-based MOF (MOF-808) NPs to encapsulate TRP and further coating it with the targeting protein transferrin (Tf). Tf is a naturally occurring iron-transport glycoprotein that binds to transferrin receptors (TfRs) highly expressed on intestinal epithelial cells [42]. By coating M@P with Tf, the nanosystem not only protects TRP from gastric acid and digestive enzymes but also exploits TfR-mediated endocytosis to enhance intestinal absorption. Following Tf modification, the final nanosystem (M@P@T) can therefore preserve TRP bioactivity and improve oral bioavailability. We selected MOF-808 as the nanocarrier due to its excellent biocompatibility, suitable pore size, and large pore volume [[37], [38], [39]]. According to Francesca Melle et al., machine learning analysis identified MOF-808 as highly biocompatible among MOF materials [40]. In addition, the pore size of MOF-808 (∼1.8 nm) can efficiently load TRP while excluding large molecular proteins such as pepsin and trypsin (∼6 nm) [41], thereby protecting the encapsulated TRP from enzymatic degradation. Through *in vitro* and *in vivo* studies, we confirmed that M@P@T NPs have excellent biocompatibility, protect TRP from degradation by gastric acid and enzymes, and achieve a high oral bioavailability of 20.5 %. Consequently, after a one-month oral administration of low-dose M@P@T NPs (200 μg kg^−1^·day^−1^) to osteoporosis model mice, therapeutic outcomes comparable to those obtained with subcutaneous TRP injections were achieved. This study provides a convenient and safe oral delivery method for TRP, showcasing its great potential for clinical application in osteoporosis patients.

## 2 Materials and methods

### 2.1 Materials

ZrOCl_2_·8H_2_O, trimesic acid, rhodamine B (Rb), and fluorescein isothiocyanate (FITC) were acquired from Anige Chemical. TRP was purchased from Rhawn Reagent, while Tf was obtained from Biosharp. Formic acid and 4′,6-diamino-2-phenylindole (DAPI) were obtained from Biotech Edge, Hefei, China. 3-[4,5-Dimethylthiazol-2-yl]-2,5-diphenyl tetrazolium bromide (MTT), fetal bovine serum (FBS), phosphate-buffered saline (PBS), and Dulbecco's modified eagle medium (DMEM) were purchased from Wuhan Kure-Bio Technology Co., Ltd. The BCIP/NBT alkaline phosphatase (ALP) color development kit, alizarin red S (ARS) staining solution, live/dead cell staining kit, and Annexin V-FITC apoptosis detection kit were procured from Beyotime Biotechnology Co., Ltd. (Jiangsu, China).

### 2.2 Preparation of MOF-808, M@P, and M@P@T

MOF-808 NPs were synthesized via a solvothermal approach. Briefly, ZrOCl_2_•8H_2_O (161.1 mg, 0.5 mmol) and 1,3,5-benzenetricarboxylic acid (35 mg, 0.167 mmol) were dissolved in a solution of 10 mL of dimethylformamide (DMF) and formic acid (1:1). The mixture was then subjected to ultrasonication for 10 min until the solution became clear and transparent, suggesting that full dissolution of all the components had been achieved. The system was left to react in an oven at 120 °C for 24 h. After cooling to room temperature, the mixture was centrifuged and washed three times with DMF, yielding MOF-808 NPs. Then, a 500 μL sample of the MOF solution was removed, and the solvent was replaced with methanol via centrifugation. The material was left to dry overnight in a vacuum dryer and then weighed for subsequent use.

To prepare M@P, the MOF solution containing 1 mg of MOF-808 was isolated, and the solvent was replaced with ddH_2_O via centrifugation. This step was followed by adding 6 mg of TRP to the solution, which was then stirred at room temperature for 12 h. The resulting mixture was centrifuged at 11,000 rpm for 10 min to yield M@P. The supernatant was collected, and the absorbance of TRP in the supernatant was measured using a UV spectrophotometer to calculate the amount of drug loaded. Subsequently, 0.5 mg of an aqueous Tf solution was added to the M@P solution with stirring for 12 h to obtain the final nanosystem, M@P@T. The MOF and TRP were stirred at various mass ratios (MOF/TRP = 1:1, 1:2, 1:4, 1:6, 1:8, and 1:10) to load the maximum amount of the drug, and the highest drug loading was achieved at a ratio of 1:6. Transmission electron microscopy (TEM) was used to examine the morphologies of the M@P@T NPs obtained after feeding different ratios of M@P and Tf (10:10, 10:5, 10:2, and 10:1), and the optimal ratio was established as 10:5.

Rb-labeled TRP (R-P) and Tf (R-T) were synthesized via N-hydroxysuccinimide (NHS) esterification reactions. FITC-labeled TRP (F-P) and Tf (F-T) were prepared using methods reported in the literature. Dual fluorescently labeled NPs (M@F-P@R-T) were synthesized using F-P and R-T, and NPs with a single fluorescent label (M@R-P) were prepared using R-P and MOF-808. M@R-P@T was prepared using R-P and unlabeled Tf.

### 2.3 Characterization

The particle sizes and zeta potentials of MOF-808, M@P, and M@P@T were measured using a Malvern Zetasizer Nano (series ZS-90). Images of their morphologies were obtained via a HITACHI H-7000FA transmission electron microscope (TEM). Elemental mapping via energy-dispersive spectroscopy (EDS) was conducted with an FEI Talos F200X instrument. Powder X-ray diffraction (PXRD) data were collected on a Rigaku MiniFlex 600 X-ray diffractometer using Cu Kα radiation (600 W). UV measurements were performed with a UV–vis spectrophotometer (UV-2600, Shimadzu, Japan). The fluorescence signals of the nanosystems were detected using a fluorescence spectrophotometer (RF-6000, Shimadzu, Japan). Confocal laser scanning microscopy (CLSM) images were obtained with a Carl Zeiss NOL-LSM 710 instrument.

### 2.4 In vitro stability

To evaluate the *in vitro* stability of the MOF-808-based nanosystem, we incubated MOF-808, M@P, and M@P@T in ddH_2_O, DMEM supplemented with 10 % FBS, and a ddH_2_O with pH = 2. The samples were placed on a shaker at 37 °C and cultivated with rotation at a speed of 120 rpm for 9 days. Changes in the particle size of the nanosystems at various time points were monitored via dynamic light scattering (DLS).

### 2.5 Release kinetics

To assess the drug release behavior from M@P and M@P@T, samples were placed in dialysis bags (MWCO = 10 kDa) and submersed in 20 mL of PBS. The samples were incubated at 37 °C on a shaker operating at 120 rpm for 24 h. At various time points (1, 2, 4, 6, 8, 12, and 24 h), a 500 μL sample was withdrawn and replaced with an equal volume of PBS to maintain a constant volume of the release medium. The amount of TRP released was determined using an enzyme-linked immunoassay (ELISA) kit, from which the cumulative drug release was calculated.

### 2.6 Cell culture

The human colon adenocarcinoma cell line Caco-2 was purchased from Procell, Wuhan, and was cultured in alpha-minimal essential medium (αMEM) supplemented with 20 % FBS and 1 % penicillin‒streptomycin. MC3T3-E1 cells were obtained from the Chinese Center for Disease Control and Prevention, Wuhan University, and were also maintained in αMEM supplemented with 10 % FBS and 1 % penicillin‒streptomycin. All cells were incubated in a cell culture incubator at 37 °C in a 5 % CO_2_ environment.

### 2.7 Cell viability assessment

Caco-2 and MC3T3-E1 cells were seeded at a density of 1 × 10^4^ cells well^−1^ in a 96-well plate. After overnight adhesion, different solutions, including MOF, M@P, and M@P@T NPs, were prepared at various concentrations (0, 31.25, 62.5, 125, 250, and 500 μg mL^−1^) in the culture medium. Then, 200 μL of each solution was added to the cells, with three replicates at each concentration. The cells were then incubated at 37 °C in a 5 % CO_2_ incubator for 24 h. Subsequently, the culture medium was replaced with a freshly prepared MTT solution (5 mg mL^−1^), and the cells were incubated for another 4 h. Finally, the MTT solution was discarded, and dimethyl sulfoxide (DMSO) (150 μL) was added to each well. After 10 min of incubation on a shaker, the absorbance at 490 nm was measured using a microplate reader, after which cell viability was calculated.

### 2.8 Live/dead cell assay

MC3T3-E1 cells were seeded in a 6-well plate at a density of 1 × 10^5^ cells well^−1^. Subsequently, MOF-808, M@P, and M@P@T were dispersed in a culture medium at a concentration of 500 μg mL^−1^ and coincubated with the cells for 3 days. The culture medium was then replaced with 500 μL of buffer containing calcein-AM and propidium iodide (PI), followed by a 15-min staining period. After washing the cells 3 times with PBS, images were obtained using an inverted fluorescence microscope.

### 2.9 Hemolysis test

Whole blood was collected from healthy female C57 mice in anticoagulant tubes, diluted with physiological saline solution, and washed 3 times with centrifugation at 2000 rpm. M@P@T at various concentrations (31.25, 62.5, 125, 250, and 500 μg mL^−1^) was combined with the mouse blood for incubation at 37 °C in a water bath for 4 h. Red blood cells (RBCs) mixed with deionized water served as the positive control, while RBCs mixed with physiological saline served as the negative control. After centrifugation, the supernatant was collected to measure the absorbance at 540 nm using a microplate reader, and the hemolysis rate of each sample was calculated based on the formula below. A hemolysis rate of less than 5 % indicates that the sample has good blood compatibility.

Hemolysis (%) = (A_sample_ − A_negative_)/(A_positive_ − A_negative_) × 100 %

### 2.10 Apoptosis assay

MC3T3-E1 cells were seeded in a 6-well plate at a density of 1 × 10^5^ cells well^−1^ and incubated overnight. MOF-808, M@P, and M@P@T were dispersed in the culture medium at a concentration of 500 μg mL^−1^ and co-incubated with the cells for 7 days. Following incubation, the cells were washed with PBS and disaggregated into single-cell suspension using trypsin. The cells were then subjected to Annexin V-FITC and PI staining via incubation in the dark for 20 min before flow cytometry observation.

### 2.11 Cellular internalization *in vitro*

To evaluate the role and mechanism of Tf in the specific uptake of nanomaterials by Caco-2 cells, we seeded Caco-2 cells in confocal dishes at a density of 1 × 10^5^ cells per dish and cultured them for 2 days. Then, a culture medium containing M@R-P or M@R-P@T was added to the dishes for coincubation with the cells for 4 h. Furthermore, to validate the mechanism of action of Tf, we preincubated cells with Tf solution for 2 h before incubation with a medium containing M@R-P@T for an additional 4 h. The medium was then discarded, and the cells were washed three times with PBS before being fixed with 4 % paraformaldehyde and stained with DAPI for 20 min. The uptake of different NPs by Caco-2 cells was observed using CLSM and quantified by flow cytometry.

Next, we further applied flow cytometry to quantitatively study the time course of cellular uptake. Caco-2 cells were placed in confocal dishes at a density of 1 × 10^5^ cells per dish for 2 days of culture, co-incubated with M@R-P@T for various durations (1, 2, or 4 h), and then washed with PBS. After digestion with trypsin to obtain a single-cell suspension, the cells were transferred to centrifuge tubes. Following centrifugation and washing with saline, the time course of cellular uptake was quantified using flow cytometry.

### 2.12 In vivo fluorescence imaging

To analyze the ability of Tf coating to enhance intestinal absorption *in vivo*, we orally administered M@R-P or M@R-P@T to overnight-fasted mice (n = 3). Then, we captured *in vivo* and ex vivo fluorescence images of the mice organs (heart, liver, spleen, lungs, kidneys, and intestines) at different time points (1, 4, 8, 12, and 24 h) using an *in vivo* optical imaging system (IVIS Spectrum, Perkin Elmer) to determine the distribution and metabolism of different NPs within the mice.

### 2.13 In vivo pharmacodynamics and pharmacokinetics studies

The pharmacokinetics and pharmacodynamics of M@P@T NPs were evaluated using the osteoporosis mice model. Mice were randomly divided into three experimental groups, with five mice per group: TRP SC, M@P, and M@P@T. M@P NPs (200 μg kg^−1^) and M@P@T NPs (200 μg kg^−1^) were administered by oral gavage, while TRP (20 μg kg^−1^) was injected subcutaneously. Serum TRP concentrations were measured using an ELISA kit. Blood samples were collected at 0, 0.5, 1, 1.5, 2, 3, 6, 8, and 12 h post-administration to determine TRP concentration. The area under the serum TRP concentration−time curve (AUC) was calculated to determine the relative bioavailability of M@P@T. The pharmacokinetic parameters of M@P@T were then compared to those of TRP SC and M@P groups.

### 2.14 ALP and ARS staining

MC3T3-E1 cells were seeded at a density of 2 × 10^4^ cells well^−1^ in a 24-well plate. After adhesion, the cells were cocultured with a medium containing MOF, M@P, or M@P@T NPs, and the medium was replaced every 3 days. A BCIP/NBT ALP color development kit and ARS staining solution were used to stain the cells after 7 and 14 days of culture, respectively. Subsequently, photographs were taken under an inverted fluorescence microscope, and semiquantitative analysis was performed with ImageJ software.

### 2.15 Quantitative real-time polymerase chain reaction (qRT‒PCR)

MC3T3-E1 cells were seeded at a density of 1 × 10^5^ cells well^−1^ in 6-well plates. After adhesion, the cells were co-cultivated with media containing MOF, M@P, or M@P@T NPs for 7 days, and the media was replaced every 3 days. After being washed with PBS, total RNA was extracted from the cells using TRIzol reagent (Invitrogen, USA). Next, the RNA was reverse transcribed into cDNA using reverse transcriptase (Vazyme Biotech Co., Ltd.), and qRT‒PCR analysis was subsequently conducted using ChamQ Universal SYBR qPCR Master Mix (Vazyme Biotech Co., Ltd.).

### 2.16 Immunofluorescence analysis

MC3T3-E1 cells were seeded in a 6-well plate at a density of 1 × 10^5^ cells well^−1^. After adhesion, the cells were cocultured for 7 days with media containing MOF, M@P, or M@P@T NPs. The culture medium was refreshed every 3 days. After culture, the cells were rinsed with PBS and fixed with 4 % paraformaldehyde. Antigens were retrieved with citric acid, and after cell membrane rupture, blocking was performed using 10 % goat serum. The cells were then incubated at 4 °C overnight with primary antibodies against mouse osteocalcin (OCN) and Runt-related transcription factor 2 (Runx2). This was followed by 1 h of incubation in the dark with an HRP-labeled secondary antibody. Nuclei were subsequently stained with DAPI. Finally, the cells were examined and imaged by CLSM.

### 2.17 Animal models

This study was approved by the Animal Ethics Committee of Wuhan University (ethics number WP20230395), and all animal experiments were conducted in accordance with the Regulations on the Management of Experimental Animals in China and the guidelines of the Committee for Animal Care and Use at Wuhan University. In this study, 6-week-old female C57 mice (purchased from Beijing HFK Bioscience Co., Ltd.) were randomly divided into five groups (Sham, OVX, M@P, M@P@T, and TRP SC). Bilateral ovariectomy was performed via a dorsal incision to establish an osteoporosis model. In the Sham group, the same volume of fat tissue was removed bilaterally. After surgery, the mice were regularly given penicillin and pain medication. All mice were housed in a comfortable environment at 22–25 °C and 25 kPa and had free access to food and water. The successful construction of the osteoporosis model was confirmed two months after surgery via micro–computed tomography (micro-CT).

### 2.18 New bone formation *in vivo*

To validate the *in vivo* efficacy of the NPs, we measured various bone parameters related to the formation of new bone and its internal structure using micro-CT (SkyScan 1276) following the ASBMR reporting standards. Each group was treated daily; the M@P and M@P@T groups were administered a water solution containing NPs (200 μg kg^−1^ TRP) via oral gavage, while the Sham and OVX groups were administered the same volume of a saline solution. The TRP SC group received a TRP solution (20 μg kg^−1^) transdermally. One month after treatment, the mice were euthanized, and the bilateral femurs were obtained. The bones were subjected to micro-CT analysis, and DataViewer and CTAn software were used to calculate and analyze the following bone parameters: the ratio of bone volume to total volume (BV/TV), trabecular thickness (Tb.Th), trabecular spacing (Tb.Sp), trabecular number (Tb.N), structure model index (SMI), and bone mineral density (BMD). The obtained images were reconstructed in three dimensions using CTVox software. The efficacy of new bone formation was assessed by comparing these parameters determined from the control and treatment groups.

After micro-CT analysis, the femur specimens were decalcified, embedded in paraffin, sectioned, and stained with hematoxylin-eosin (H&E) and Masson's trichrome for histopathological analysis.

To further assess the expression of osteogenesis-related proteins, immunohistochemical staining for Runx2, osteopontin (OPN), OCN, and collagen I (COL1) was performed on the femurs according to previously reported protocols, and semiquantitative analysis was carried out using ImageJ.

### 2.19 Evaluation of *in vivo* safety

To evaluate whether the MOF-808-based nanosystem induces cumulative organ toxicity, the major organs (heart, liver, spleen, lungs, kidneys, and intestines) of the mice were collected after treatment, fixed in paraformaldehyde, and embedded in paraffin. The tissues were then sectioned into thin slices and stained with H&E. Morphological changes in the tissues were observed using a microscope.

Additionally, to assess the blood compatibility of long-term MOF treatment, we orally administered either an aqueous MOF solution (400 μg kg^−1^) or an equivalent volume of ddH_2_O to the mice (n = 5) daily. After 30 days of continuous administration, whole blood samples were collected from the mice, and routine blood examinations were performed.

### 2.20 Statistical analysis

All experimental data were collected from three independent experiments and are presented as the mean ± standard deviation (mean ± SD). GraphPad Prism 8.4.1 was used for the statistical analysis, and two-tailed Student's t-tests were used for between-group comparisons. *P* < 0.05 (∗) was considered to indicate a statistically significant difference, while *P* < 0.01 (∗∗) and *P* < 0.001 (∗∗∗) were considered to indicate highly statistically significant differences.

## 3 Results and discussion

### 3.1 Preparation and characterization of MOF-based oral delivery nanosystems

**Fig. 1:**
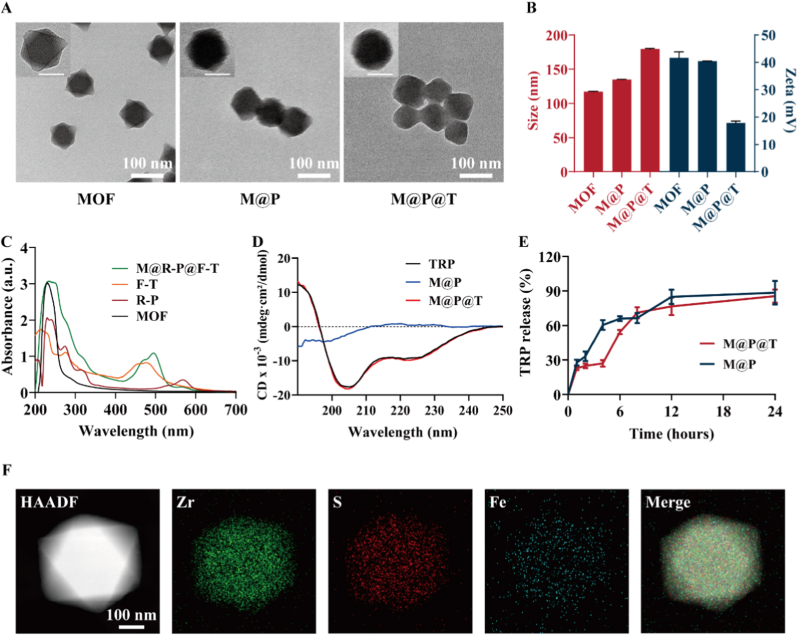
Characterization of MOF-808, M@P, and M@P@T. (A) TEM images of the MOF-808-based nanosystems. Scale bar, 100 nm (Inset scale bar: 50 nm). (B) Hydrodynamic sizes and zeta potentials of different preparations dispersed in ddH_2_O determined by DLS. (C) UV–vis absorption spectra of MOF-808, R-P, F-T and M@R-P@F-T. (D) Circular dichroism (CD) spectra of TRP from the normal control group (dissolved in PBS), M@P group, and M@P@T group (both incubated in a simulated gastric acid environment (pH = 2) at 37 °C for 2 h). (E) *In vitro* drug release kinetics from M@P and M@P@T in PBS (pH = 7.4). (F) EDS elemental mapping analyses of M@P@T. Scale bar, 100 nm.

The preparation processes for MOF-808, M@P, and M@P@T NPs are illustrated in Scheme 1A. MOF-808 was synthesized by a simple solvothermal method, and its regular octahedral morphology can easily be discerned from the TEM images (Fig. 1A). For the effective oral delivery of peptides/proteins, NPs should ideally have an appropriate size to facilitate diffusion through the mucus layer and subsequent absorption by intestinal epithelial cells. The pore size of the mucus layer has been reported to be ∼200 nm [43,44]. With a hydrodynamic diameter of 117.2 ± 0.2 nm (Fig. 1B), MOF-808 can easily navigate through the mucus layer. The zeta potential of MOF-808 was +42 mV, which would enable the NPs to capture negatively charged peptides and proteins, including TRP, through electrostatic interactions at neutral pH. After loading TRP, the morphology of the M@P NPs became rounder, the edge angles became blunted, the particle size increased slightly to 134.9 ± 0.23 nm, and the zeta potential decreased to +40 mV. Then, the larger protein molecule Tf was added to the reaction system and became conjugated to the surface of M@P via simple electrostatic interactions. All reactions were performed at room temperature to better preserve the bioactivity of the encapsulated TRP. After Tf modification, the surface of the M@P@T NPs was rounder than that of the M@P NPs. The morphology of the M@P@T NPs gradually transitioned from octahedral to spherical, and TEM showed a core-shell structure composed of MOF-808 internally with Tf coating on the outside. Following Tf modification, the particle size noticeably increased to 179.8 ± 3.4 nm, but was still smaller than the pore size of the mucus layer (∼200 nm). The zeta potential of the M@P@T NPs decreased further to +18 mV.

Subsequently, we used rhodamine B to label TRP (denoted as R-P) and labeled Tf with FITC (denoted as F-T) to prepare a dual fluorescently labeled MOF nanosystem (M@R-P@F-T). As shown in Fig. 1C, the UV–vis absorption peaks of rhodamine B and FITC could be discerned concurrently within M@R-P@F-T, validating the successful loading of TRP and Tf onto MOF-808. The fluorescence spectra results (Fig. S1) further confirmed this conclusion. The powder X-ray diffraction (PXRD) study (Fig. S2) revealed that the pattern of MOF-808 NPs was consistent with the calculated pattern from MOF-808 single crystals and the patterns of both M@P and M@P@T NPs, suggesting that MOF-808 nanocarrier maintained its crystallinity throughout the TRP loading and Tf decoration processes. Elemental mapping was performed to corroborate the presence of each component within the M@P@T NPs, with Zr indicating MOF-808, S indicating protein, and Fe indicating Tf. As illustrated in Fig. 1F, Zr, S, and Fe were evenly distributed across the NPs. Notably, S exhibited a slightly broader distribution than Zr, while Fe exceeded Zr and S atoms in spatial dispersion. This result suggests that TRP was successfully loaded into MOF-808 and that Tf was uniformly dispersed across the surface of M@P. To further investigate potential bond changes during the loading of TRP and Tf, FT-IR spectra of MOF-808, M@P, and M@P@T were recorded (Fig. S3). After TRP loading, new absorption bands appeared at approximately 1630 cm^−1^ and 1550 cm^−1^, which are characteristic of peptide backbone vibrations. These signals became more pronounced in M@P@T, confirming the successful loading of TRP and Tf. Importantly, the characteristic peaks of MOF-808 remained preserved, indicating that no significant covalent bond disruption occurred and that the framework structure of MOF-808 remained intact during the loading process. Furthermore, we performed thermogravimetric analysis (TGA) to evaluate the thermal stability of the nanosystems (Fig. S4). The TGA curves showed greater weight loss between 150 °C and 400 °C for M@P and M@P@T than for MOF-808, primarily ascribed to the degradation of TRP and Tf. The above results collectively demonstrate the successful construction of M@P@T NPs.

Meanwhile, we also used UV spectroscopy to further evaluate the drug loading capacity of MOF-808 by measuring the absorbance of the supernatant after mixing MOF-808 and TRP at different mass ratios (1:1, 1:2, 1:4, 1:6, and 1:8). At a mass ratio of 1:6, the drug loading capacity of MOF-808 reached 56 wt% (Fig. S5), which is significantly greater than that of most reported nanocarriers [45]. To quantitatively assess Tf loading, the drug loading content (DLC) was determined at different M@P:Tf mass ratios (Fig. S6). The DLC increased with higher Tf feeding ratios and reached the maximum at 10:5.

Additionally, we monitored the change in the size of the nanosystems in different solvents to assess the *in vitro* stability of MOF-808. After MOF-808, M@P, and M@P@T NPs were dispersed in a simulated gastric pH environment (dilute HCl, pH = 2) for 9 days, no discernible changes in particle size were observed (Fig. S7), which confirmed the good acid stability of the MOF-808-based NPs. However, after 9 days of incubation in DMEM supplemented with 10 % FBS, M@P without the Tf coating showed a noticeable decrease in particle size (Fig. S8), which could be ascribed to the start of MOF-808 decomposition. These findings indicate that the Tf coating on the MOF-808 nanosystem can delay its decomposition, enhancing the stability of the nanosystem in the acidic environment of the stomach. To further evaluate structural stability, TEM was used to monitor the morphological changes of MOF-808, M@P, and M@P@T under phosphate-rich conditions (Fig. S9). After 4 h of incubation, MOF-808 displayed noticeable morphological changes, whereas M@P and M@P@T maintained well-defined nanostructures. At 8 h, MOF-808 underwent severe degradation with loss of architecture, while M@P@T largely retained its integrity. These results confirm that peptide loading and Tf coating markedly enhance the stability of the nanosystem against phosphate-induced degradation, which is essential for achieving controlled and sustained TRP release under physiological conditions.

Next, we incubated M@P and M@P@T in a simulated gastric acid environment (pH = 2) at 37 °C for 2 h to further evaluate the protective effect of Tf. Subsequently, PBS was used to lyse the MOF NPs and release the loaded TRP. The secondary structure of TRP was then analyzed by circular dichroism (CD) spectroscopy. The results showed that the TRP in the M@P@T group, which was coated with Tf, retained its secondary structure and was similar to that of the control group (Fig. 1D), while the TRP in the M@P group exhibited significant structural degradation. These findings confirm that Tf plays a protective role in preserving the secondary structure of TRP in the gastric acid environment.

To be an effective oral peptide delivery system, M@P@T NPs not only provide sufficient protection of TRP in acidic environments but also need to achieve controllable release of TRP from the acid-resistant nMOFs under physiological conditions. As depicted in Fig. 1E, under phosphate-rich physiological conditions (PBS), the rate of TRP release gradually increased over time, exceeding 80 % after 12 h. This sustained release is mechanistically attributed to phosphate-triggered degradation of the MOF-808 framework, in which phosphate ions gradually disrupt Zr–O coordination, leading to controlled TRP liberation. In the initial 7 h, M@P@T exhibited a slower release rate than M@P, likely because the Tf coating delayed phosphate access to the MOF surface, thereby extending the release period. At 24 h, both formulations showed nearly 90 % drug release. These findings indicate that phosphate-driven structural degradation underlies the controlled and sustained release behavior of M@P@T.

### 3.2 *In vitro* biocompatibility analysis

**Fig. 2:**
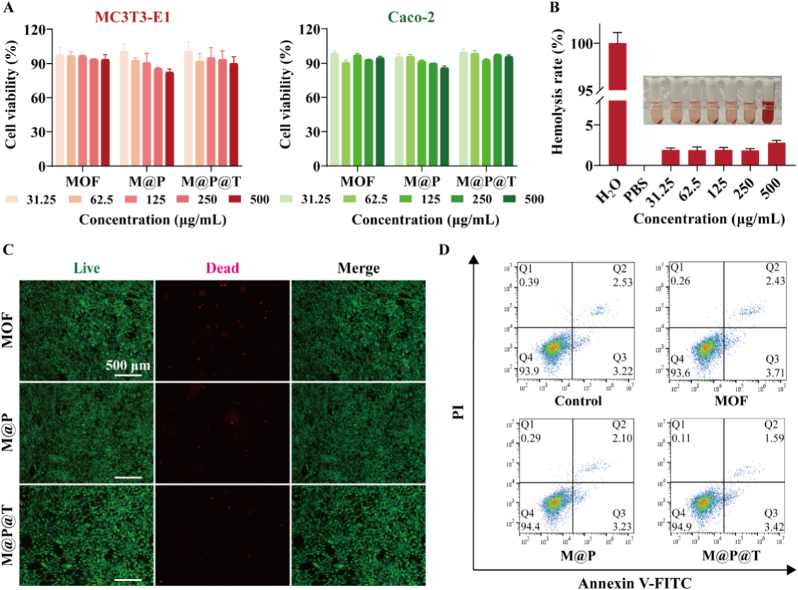
*In vitro* biocompatibility of the MOF-808-based nanosystems. (A) Assessment of the viability of MC3T3-E1 and Caco-2 cells after treatment with the MOF-808-based nanosystems *in vitro*. (B) Hemolysis test with M@P@T. (C) Live/dead staining images after treatment with the MOF-808-based nanosystems. Scale bar, 500 μm. (D) Flow cytometry results with the MOF-808-based nanosystems.

Biosafety is a fundamental prerequisite of functional nanosystems for biomedical applications. In this study, to evaluate the biosafety of the M@P@T NPs, we first conducted MTT assays. As shown in Fig. 2A, the NPs were co-incubated with two cell lines (MC3T3-E1 and Caco-2) for 1 day, and the optical density (OD) values of the three groups of NPs gradually decreased as the drug concentration increased. Notably, even at a nanosystem concentration of 500 μg mL^−1^, the cell survival rate remained above 80 % across all groups, demonstrating the good biocompatibility of the MOF-808 nanosystem. Furthermore, results from the 3-day CCK8 assay (Fig. S10) demonstrated that cell viability increased over the three days for all groups, consistently staying above 90 %, which is comparable to the control group. No significant cytotoxicity was observed, suggesting that the loading of TRP and Tf did not adversely affect cell viability. Additionally, we assessed the blood compatibility of the MOF-808 nanosystem by co-incubating M@P@T NPs with erythrocytes for 4 h (Fig. 2B). The results showed that the hemolysis rate was less than 5 % even when the material concentration reached 500 μg mL^−1^. In subsequent live/dead staining assays, we assessed the viability of MC3T3-E1 cells after 3 days of co-incubation with the MOF-808 nanosystem. As shown in Fig. 2C, almost all the MC3T3-E1 cells in the three groups were alive (green), and the number of dead cells (red) was essentially negligible, indicating that the nanosystem caused very little damage to the cells. To further assess cellular morphology, cytoskeleton and nuclear staining were performed (Fig. S11). Cells treated with MOF, M@P, and M@P@T showed morphology comparable to the control, with intact F-actin filaments and nuclei, and no signs of cytoskeletal disruption. When the co-incubation time was extended to 7 days, apoptosis analysis by flow cytometry showed relatively high cell survival rates and negligible levels of apoptosis in all treatment groups (Fig. 2D). Therefore, these results collectively demonstrate that the MOF-808-based nanosystem exhibits good biosafety and does not adversely affect the survival or growth of osteoblasts or intestinal epithelial cells.

### 3.3 Tf-mediated augmentation of intracellular and *in vivo* absorption

**Fig. 3:**
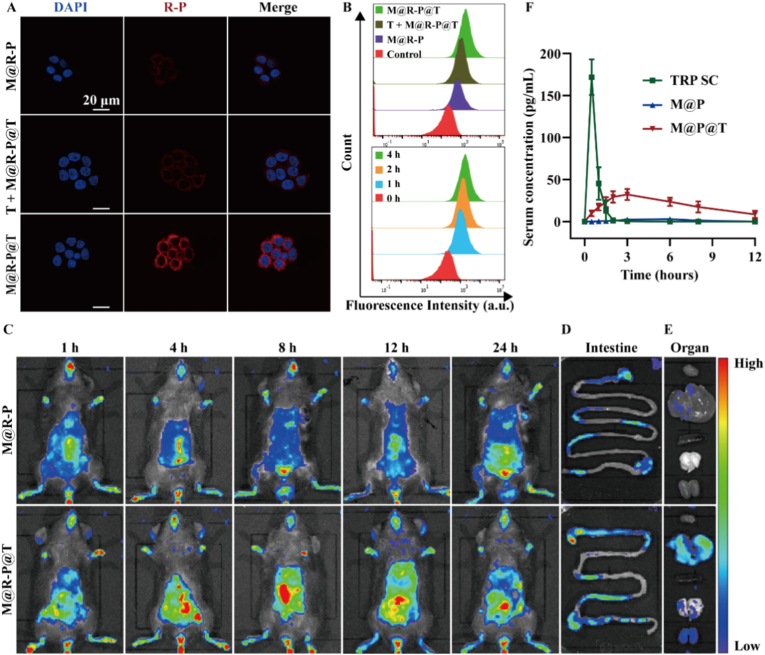
Tf-mediated augmentation of intracellular and *in vivo* absorption. (A) CLSM images of Caco-2 cell monolayers incubated with MOF-808-based nanosystems for 4 h. Blue indicates the nuclei and red indicates TRP. Scale bar, 20 μm. (B) Quantitative analysis of cellular uptake of different formulations at 4 h and M@P@T at various time points determined by flow cytometry. (C) Fluorescence distribution and intensity of R-P *in vivo* at different time points after the oral administration of different NPs. *Ex vivo* fluorescence images of the intestine (D) and images of heart, liver, spleen, lung, and kidney (from top to bottom) (E) of the mice captured 12 h after the oral administration of NPs. (F) Serum TRP concentrations measured after free TRP (SC) and the administration of the M@P and M@P@T to osteoporotic mice through oral gavage (n = 5 biologically independent animals, means ± SDs). SC, subcutaneous injection. (For interpretation of the references to color in this figure legend, the reader is referred to the Web version of this article.)

At present, due to the structural and functional similarities between human colon adenocarcinoma (Caco-2) cells and small intestinal epithelial cells, Caco-2 cells are extensively used in studies on drug absorption through intestinal cells [46,47]. Hence, this study sought to use Caco-2 cells as an *in vitro* cell model to investigate whether Tf could facilitate the transcytosis of the MOF-808 nanosystem across the intestinal epithelium via TfR-mediated endocytosis, thereby bolstering the oral absorption of MOF-808. Using CLSM and flow cytometry, qualitative and quantitative studies of Caco-2 cell uptake were performed. Fig. 3A shows that after the same incubation duration, the M@P@T group had a stronger fluorescence signal than the M@P group, indicating that the Tf coating considerably enhanced the cellular uptake of the MOF-808 nanosystem. Additionally, when the cells were pre-incubated with free Tf for 2 h, the fluorescence signal in the M@P@T group decreased to a level comparable to that in the M@P group. This could be caused by free Tf binding to TfR on the cellular membrane before the addition of M@P@T, thereby inhibiting the cellular uptake of M@P@T. These data validate that the uptake of M@P@T by Caco-2 cells was primarily mediated by Tf-TfR interactions. Additionally, the flow cytometry results shown in Fig. 3B confirmed these findings, with the quantitative cellular uptake of M@P@T being approximately 2.2 times greater than that of M@P. Moreover, TfR-mediated cellular uptake was time-dependent, as cellular uptake progressively increased over time and reached approximately 63.7 % within 1 h. This indicates that the Tf-coated MOF-808 nanosystem possesses rapid and efficient cellular uptake capabilities.

To further elucidate the gut absorption and systemic distribution of M@P@T in mice, we employed a small animal *in vivo* imaging system. Rhodamine B-labeled TRP was used in this study, and the intensity and distribution of the fluorescence signal from R-P were observed at various intervals after administration. According to the imaging results (Fig. 3C and S12A), a strong fluorescence signal was noted in the M@R-P@T group as early as 4 h after administration, which peaked at 8 h and was still prominently observable after 12 h. At each time point, the fluorescence intensity of M@R-P@T was greater than that of the control group (M@R-P). These results suggest that Tf binds to TfR on intestinal epithelial cells to promote the uptake of the MOF-808 nanosystem and prolong the residence time of the nanosystem in the intestine.

Subsequently, fluorescence images of the intestines (Fig. 3D) and organs (Fig. 3E and S12B) excised 12 h after the administration of different treatments revealed that compared to the M@R-P group, the M@R-P@T group had a stronger and broader intestinal fluorescence signal. Notably, an identical phenomenon was observed in the liver, which can be attributed to the portal vein carrying M@R-P@T absorbed in the intestines to accumulate in the liver. This confirmed that the Tf-coated nanosystem enhances intestinal uptake. In addition, there was almost no fluorescence enrichment in any organs other than the kidneys, which indicates that after M@P@T NPs are absorbed by the body via the intestine, they are mainly metabolized by the kidneys and will not accumulate in other organs and cause organ toxicity. Hence, our results indicate that the Tf coating significantly enhanced NP uptake by intestinal epithelial cells both *in vivo* and *in vitro*, thus facilitating the efficient oral delivery of TRP.

We evaluated the pharmacokinetics and bioavailability of M@P@T *in vivo* by administering M@P and M@P@T (200 μg kg^−1^) to osteoporotic mice via oral gavage, while free TRP (20 μg kg^−1^) was administered subcutaneously (SC) as a control. Serum TRP concentrations were measured using a TRP ELISA kit. As illustrated in Fig. 3F, the plasma concentration of TRP in the TRP SC group rapidly peaked at 0.5 h post-injection but declined sharply, returning to the baseline level within 2 h. In contrast, the M@P@T group maintained a relatively higher and more stable plasma concentration of TRP for up to 12 h, whereas the M@P group consistently maintained low TRP levels. Pharmacokinetic parameters derived from the serum TRP concentration–time curves are summarized in Table S2. Notably, the area under the curve (AUC) of M@P@T was 240.1 pg h·mL^−1^, and its relative bioavailability was 20.5 %, which was 13.6-fold higher than that of the M@P group (1.5 %). These findings indicate that the Tf-coated nMOF system (M@P@T) significantly enhances intestinal epithelial cell uptake and improves bioavailability. Additionally, the sustained release properties of M@P@T effectively prolong the duration of action of TRP *in vivo*, showcasing its potential as an ideal oral delivery system for osteoporosis treatment.

### 3.4 *In vitro* osteogenic differentiation

**Fig. 4:**
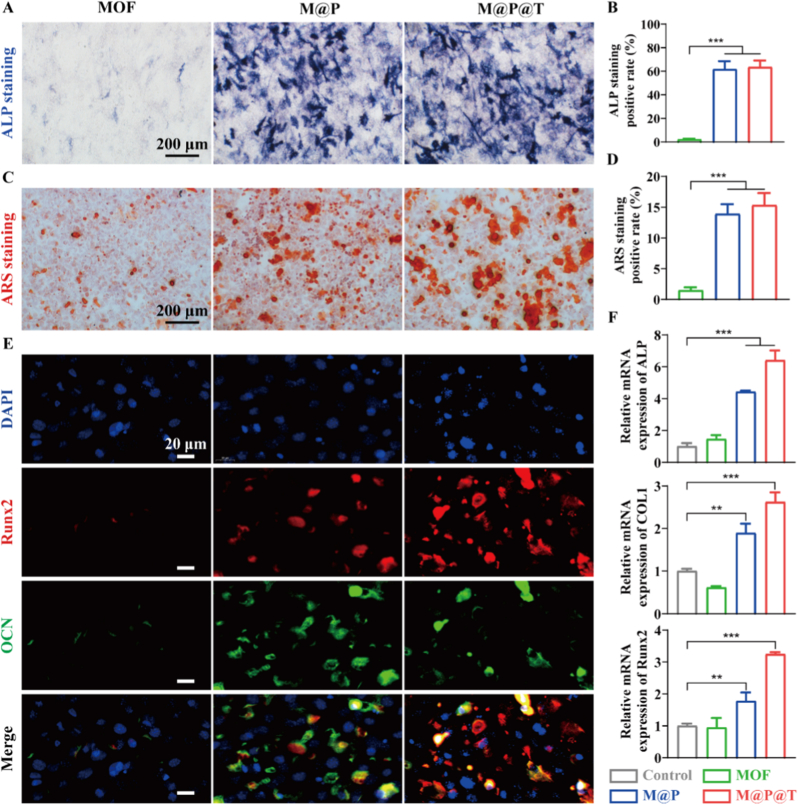
M@P@T promoted the osteogenic differentiation and mineralization of MC3T3-E1 cells *in vitro*. (A) ALP staining images of MC3T3-E1 cells and (B) the corresponding semi-quantitative analysis after 7 days of culture. (C) ARS staining images of MC3T3-E1 cells and (D) the corresponding semiquantitative analysis after 14 days of culture. (E) Representative immunofluorescence images of Runx2 (red) and OCN (green), with the nuclei shown in blue. (F) Relative mRNA expression levels of osteogenesis-related genes, including ALP, COL1 and Runx2, in MC3T3-E1 cells cultured with various NPs determined via qRT–PCR analysis on day 7. (For interpretation of the references to color in this figure legend, the reader is referred to the Web version of this article.)

Next, we treated MC3T3-E1 cells with M@P@T NPs to investigate their osteogenic effects. ALP is a critical early biological marker of osteogenesis [48]. Thus, ALP staining was performed on day 7. The qualitative and semiquantitative results (Fig. 4A and B) showed that compared with MOF alone, M@P and M@P@T NPs significantly increased ALP expression, suggesting that the TRP-loaded MOF nanosystems possess superior osteogenic bioactivity, which could benefit from the suitable release of TRP under physiological conditions. ARS staining, which is a pivotal indicator of late osteogenic differentiation, was utilized to detect extracellular matrix (ECM) calcium deposits. On day 14, the qualitative and semiquantitative analyses (Fig. 4C and D) demonstrated that there were notably more calcium deposits in the M@P and M@P@T groups than in the MOF group, a finding that aligns with the ALP activity measurements. Moreover, the M@P@T group showed effects similar to those of the M@P group, indicating that after 3 days of incubation under physiological conditions, M@P@T and M@P released nearly the same amounts of TRP. Thus, the Tf coating did not affect the release amount of TRP, providing a robust indication for *in vivo* treatment.

According to the literature, COL1, OPN, and OCN make significant contributions to the composition of the bone ECM [49], while Runx2 plays a vital role in modulating the expression of osteogenesis-related genes. We further elucidated the genetic mechanisms through which M@P@T promotes osteogenic differentiation by performing qRT‒PCR to measure the expression levels of osteogenesis-related genes in MC3T3-E1 cells. As shown in Fig. 4F, the ALP, Runx2, and COL1 expression levels in the M@P and M@P@T groups were markedly higher than those in the MOF group, suggesting that TRP functions primarily by upregulating osteogenic markers within cells. Immunofluorescence staining (Fig. 4E and S12C) revealed that the amounts of osteogenic proteins (Runx2 and OCN) secreted by the MC3T3-E1 cells in the M@P@T and M@P groups surpassed those secreted by the cells in the MOF group, further supporting the role of M@P@T in promoting osteogenesis. Collectively, these results emphasize the pronounced role of M@P@T in fostering osteogenic differentiation in MC3T3-E1 cells, which was predominantly achieved through the upregulation of the ALP, Runx2, COL1, and OCN genes to facilitate bone formation.

### 3.5 Assessment of bone formation *in vivo*

**Fig. 5:**
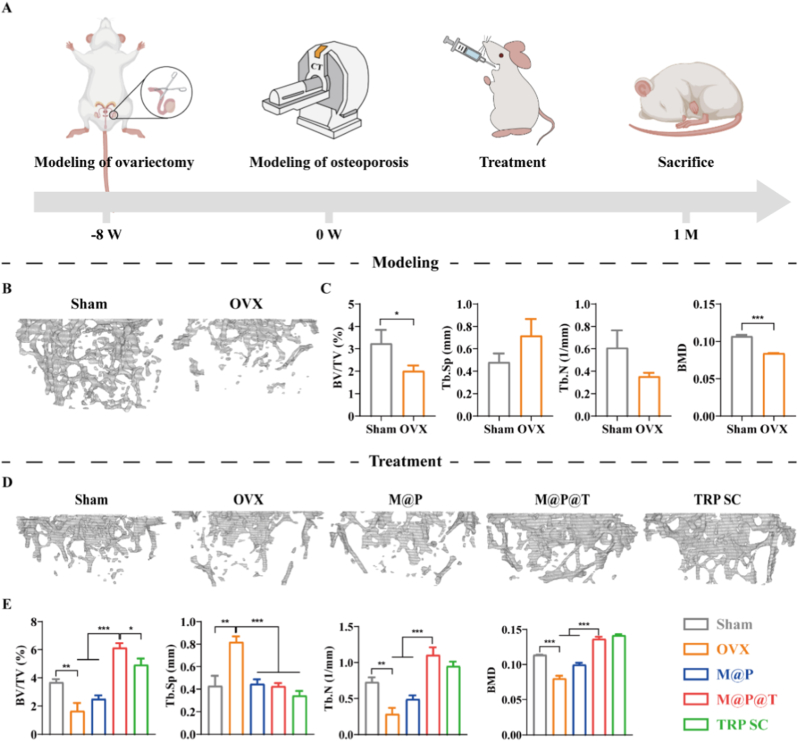
Assessment of bone formation *in vivo*. (A) Schematic of osteoporosis model building. (B) 3D reconstructed images of trabecular tissue from sham-operated mice (Sham, left) and ovariectomized mice (OVX, right). (C) Quantitative analysis of bone parameters, including bone volume/total volume (BV/TV), trabecular separation (Tb.Sp), trabecular number (Tb.N) and bone mineral density (BMD) in Sham and OVX mice 8 weeks after surgery. (D) 3D reconstructed images of trabeculae and (E) the corresponding quantitative analysis of bone parameters of OVX mice after one month of treatment with different preparations.

Given the excellent biocompatibility and pharmacokinetic characteristics of the M@P@T NPs, we further explored their ability to promote bone formation in mice. To determine the regenerative capacity of M@P@T NPs in an osteoporotic environment *in vivo*, we first established an osteoporosis model by surgically removing the bilateral ovaries of the mice (Fig. 5A). Eight weeks after surgery, the status of the femoral metaphysis trabeculae was evaluated via micro-CT. The trabecular bone parameters and three-dimensional reconstructed images (Fig. 5B and C) revealed significant differences between the trabeculae in the Sham and OVX groups eight weeks after surgery. Notably, trabecular bone volume and density in the OVX group were significantly reduced, confirming the successful establishment of the osteoporosis model.

After the successful model establishment, M@P and M@P@T NP solutions were administered to the model mice by gavage once daily, and free TRP was administered via subcutaneous injection. The micro-CT results obtained after one-month treatment (Fig. 5D) revealed that the volume, number, and density of bone trabeculae in the mice from the M@P@T group increased significantly, while the degree of trabecular separation was markedly decreased, and these results were similar to those obtained after the subcutaneous injection of TRP. Three-dimensional reconstruction images of the bone trabecula can vividly and accurately demonstrate the effects of bone repair. As shown in Fig. 5E, the reconstructed images of the bone trabeculae show various degrees of new bone formation in response to oral treatment with M@P and M@P@T as well as the subcutaneous injection of TRP, which was consistent with our expectations. Furthermore, the M@P@T and the TRP SC groups exhibited more new bone formation than the other groups, indicating that the oral administration of M@P@T can produce a therapeutic effect in osteoporotic mice that is comparable to that of a subcutaneous injection of TRP.

### 3.6 Histological evaluation of osteogenesis

**Fig. 6:**
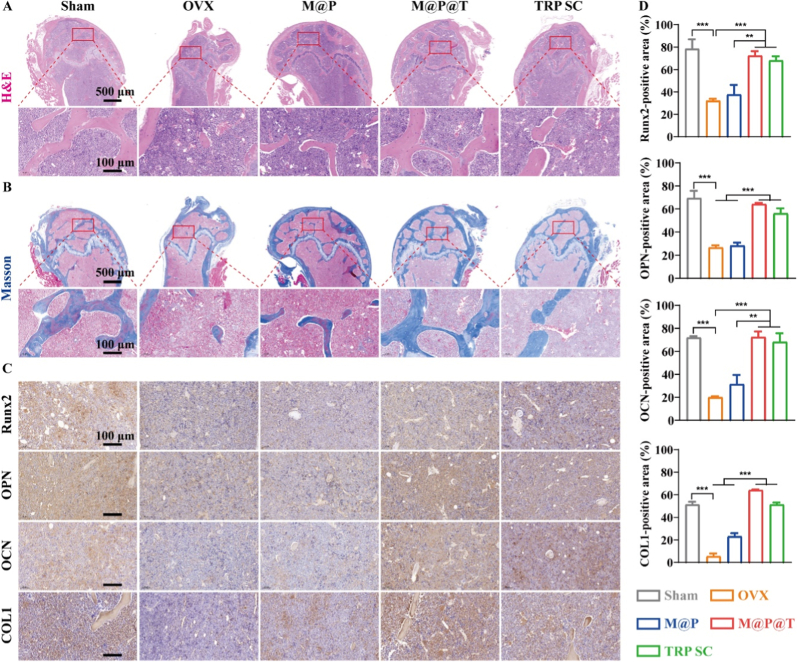
Histological evaluation of osteogenesis *in vivo*. Representative images of H&E staining (A) and Masson staining (B) after one month of treatment with different preparations. (C) Representative immunohistochemical staining images and (D) semiquantitative analysis of the expression levels of osteogenesis-related proteins, including Runx2, OPN, OCN, and COL1.

Hematoxylin and eosin (H&E) staining, along with Masson's trichrome staining, are widely used histological methods for tissue analysis. H&E staining effectively highlights cell nuclei and cytoplasm, which are essential for examining the morphology and distribution of osteoblasts in bone tissue. In contrast, Masson's trichrome staining specifically targets collagen fibers, which appear blue, thereby providing a clear view of their distribution within the bone. Using H&E and Masson's trichrome staining, we evaluated osteogenesis in the femoral metaphyses of the mice. H&E staining (Fig. 6A) revealed that, compared with the OVX group, the M@P@T group displayed much more trabecular bones with a significantly greater volume, similar to the findings from the TRP SC and Sham groups. Moreover, Masson's trichrome staining (Fig. 6B) demonstrated that the M@P@T group had more abundant and thicker collagen fibers than the other groups, indicating increased osteogenesis. These findings are consistent with the previously mentioned micro-CT results.

To further clarify the potential mechanisms underlying the promotion of osteogenesis by M@P@T at the tissue level, we conducted immunohistochemical staining analyses of Runx2, OPN, OCN, and COL1 in sliced sections of mouse femurs. As depicted in Fig. 6C and D, the M@P@T group exhibited a greater number of positively stained areas (indicated by brown coloration) compared to the other groups. This suggests that M@P@T enhances the expression of osteogenesis-related proteins. These *in vivo* immunohistochemical results align with the outcomes observed in our *in vitro* cellular experiments.

### 3.7 Long-term biosafety of M@P@T *in vivo*

**Fig. 7:**
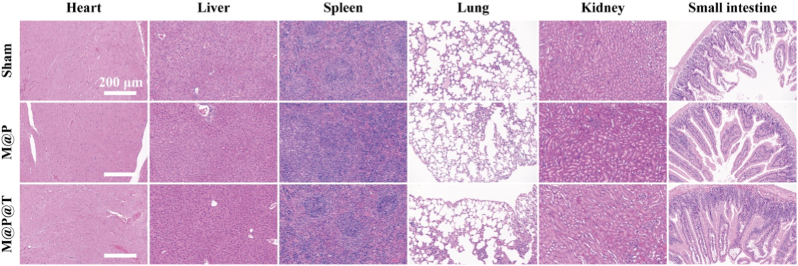
H&E staining images of the heart, liver, spleen, lung, kidney and small intestine harvested after one-month of different treatments. Scale bar, 200 μm.

The long-term safety of treatment methods for osteoporosis, a chronic disease, is paramount. Thus, after one month of treatment, we performed H&E staining on sections of the major organs from the treated mice. The results revealed no obvious pathological damage in the heart, liver, spleen, lung, kidney, or intestine (Fig. 7). The villi structure of the small intestine remained intact, indicating that after long-term oral administration, the MOF-808 nanosystem did not accumulate in the organs, cause toxicity, or damage intestinal epithelial cells.

Based on these findings, we further assessed the biosafety of the MOF-808 nanocarrier under high-dose conditions by administering doses at 100x, 500x, and 1000x the standard concentration over one month. We conducted a comprehensive hematological analysis and assessed liver and kidney function parameters to evaluate potential systemic toxicity. The hematological results (Fig. S13) —including counts of white blood cells, red blood cells, hemoglobin, platelets, lymphocytes, and neutrophils—remained within normal physiological ranges across all dose groups, indicating an absence of hematological toxicity. Similarly, liver function markers such as alanine aminotransferase (ALT) and aspartate aminotransferase (AST), along with renal function indicators such as blood urea nitrogen (BUN) and creatinine (CR), showed no significant deviations from the baseline values (Fig. S14), confirming the absence of hepatotoxicity and nephrotoxicity, even after prolonged treatment with high doses. Furthermore, a histopathological examination using H&E staining of major organs—including the heart, liver, spleen, lung, kidney, and intestine—revealed no structural abnormalities in any of the dose groups (Fig. S15). The tissue integrity was preserved, with no evidence of inflammatory cell infiltration, cellular degeneration, or fibrosis. Notably, the intestinal villus structure remained intact, showing no signs of erosion or epithelial damage, supporting the nontoxicity of the nanosystem to the gastrointestinal tract even at elevated doses. Collectively, these findings show that the MOF-808 nanosystem exhibits excellent biocompatibility and maintains a favorable safety profile under long-term high-dose administration, underscoring its potential for extended oral use in osteoporosis treatment.

## 4 Conclusion

In conclusion, we have successfully developed an oral TRP delivery system based on Tf-engineered acid-resistant nMOF (M@P@T NPs) for the effective treatment of osteoporosis. This M@P@T nanosystem features high drug loading, good stability in the gastrointestinal tract, and controllable drug release in the bloodstream. Both *in vivo* and *in vitro* uptake studies demonstrated that M@P@T can protect TRP from harsh gastrointestinal environments and effectively facilitate intestinal absorption via the TfR-mediated pathway. Furthermore, the *in vivo* and *in vitro* osteogenesis experiments revealed that the oral administration of low-dose M@P@T to osteoporosis model mice promotes osteoblast differentiation and new bone formation by increasing the expression of osteogenesis-related genes, achieving a therapeutic outcome comparable to that of subcutaneous TRP injections. Importantly, no organ damage was observed in the mice following long-term treatment, highlighting the long-term biosafety of this nanosystem. Therefore, we believe that M@P@T is a highly biocompatible nanosystem capable of efficient oral TRP delivery, which could improve long-term treatment adherence among osteoporosis patients and present significant transformative potential for clinical application. Future research will aim to validate the long-term biosafety and therapeutic effect of the MOF-based oral delivery nanosystem in larger animal models.

## CRediT authorship contribution statement

**Renxiong Wei:** Writing – review & editing, Writing – original draft, Methodology, Investigation, Funding acquisition, Data curation. **Sang Hu:** Writing – review & editing, Writing – original draft, Methodology, Investigation, Formal analysis, Data curation. **Jiazhi Wang:** Writing – review & editing, Methodology, Investigation, Data curation. **Qingjian Lei:** Methodology, Investigation. **Zhiyu Jiang:** Methodology, Investigation. **Bo Wang:** Methodology, Investigation. **Haixia Yang:** Methodology, Investigation. **Feifei Yan:** Writing – review & editing, Supervision, Project administration, Conceptualization. **Lin Cai:** Writing – review & editing, Supervision, Project administration, Conceptualization. **Jian Tian:** Writing – review & editing, Writing – original draft, Supervision, Project administration, Funding acquisition, Conceptualization.

## Ethics approval and consent to participate

The Animal Ethics Committee of Wuhan university approved all animal experiments in this project (ethics number WP20230395).

## Consent for publication

All authors consented to publish the article.

## Funding

This work was supported by the following funding: 10.13039/501100001809National Natural Science Foundation of China no. 10.13039/501100001809NSFC 22271222 (J.T.), 10.13039/501100016359Zhongnan Hospital of Wuhan University Translational Medicine and Interdisciplinary Research Joint Fund no. ZNJC 202220 (R.W.).

## Declaration of competing interest

The authors declare that they have no known competing financial interests or personal relationships that could have appeared to influence the work reported in this paper.

## Data Availability

Data will be made available on request.
