# Supplementary material for: Oral delivery of teriparatide utilizing biocompatible transferrin-engineered MOF nanoparticles for osteoporosis therapy
Source: Mater Today Bio. 2025 Sep 15;35:102318. doi: 10.1016/j.mtbio.2025.102318 (PMC12481929; doi:10.1016/j.mtbio.2025.102318)
Supplement: Multimedia component 1 [file mmc1.docx]

Supplementary Materials

**Oral delivery of teriparatide utilizing biocompatible** **transferrin-engineered MOF nanoparticles for** **osteoporosis therapy**

*Renxiong Wei^#a^, Sang Hu^#a^, Jiazhi Wang^#a^, Qingjian Lei^a^, Zhiyu Jiang^a^, Bo Wang^a^, Haixia Yang^a^, Feifei Yan^*a^, Lin Cai^*a^ and Jian Tian^*a, b^*

^a^ Department of Spine Surgery and Musculoskeletal Tumors, Zhongnan Hospital of Wuhan University, School of Pharmaceutical Sciences, Wuhan University, Wuhan 430071, China.

^b^ State Key Laboratory of Metabolism and Regulation in Complex Organisms, College of Life Sciences, Wuhan University, Wuhan 430071, China.

^#^ These authors have contributed equally to this work.

* Correspondence should be addressed to:

*E-mail: yanfeifei0120@whu.edu.cn (F.Y.); orthopedics@whu.edu.cn (L.C.); jian.tian@whu.edu.cn (J. T.)

**Table S1. Primer sequences used in qRT-PCR analysis.**

| **Genes** | **Primers (F, forward; R, reverse; 5’-3’)** |
| --- | --- |
| Mouse-GAPDH | F: TCAACGGCACAGTCAAGG |
|  | R: TTAGTGGGGTCTCGCTCC |
| Mouse-Runx2 | F: CATCCCAGTATGAGAGTAGGTGT |
|  | R: GCTCAGATAGGAGGGGTAAGAC |
| Mouse-COL1 | F: CTGACTGGAAGAGCGGAGAG |
|  | R: CGGCTGAGTAGGGAACACAC |
| Mouse-ALP | F: GGTATGGGCGTCTCCACAGT |
|  | R: GCCCGTGTTGTGGTGTAGCT |


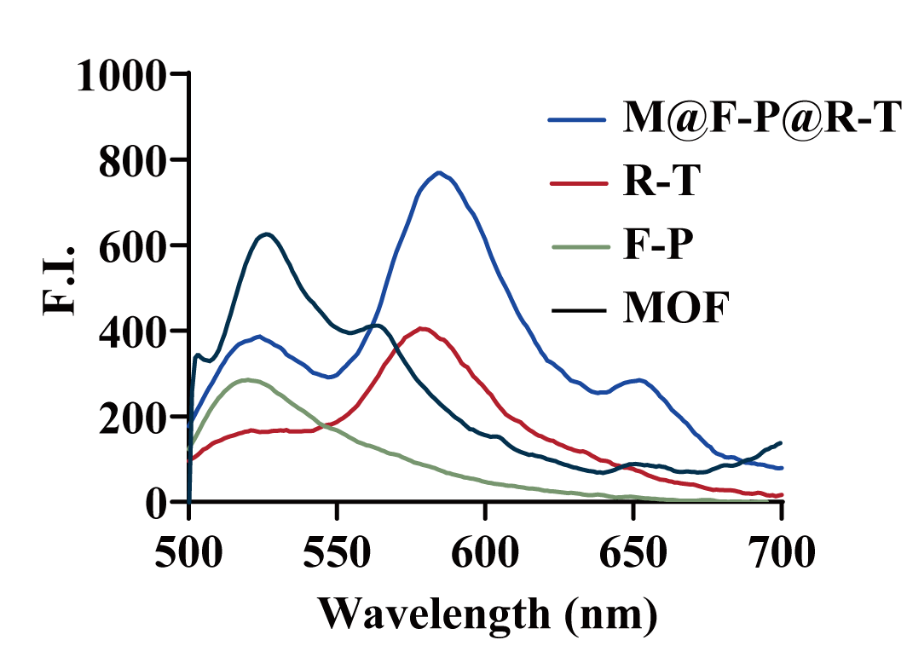


**Figure S1.** Emission spectra of different preparations with excitation at 450 nm. F.I., fluorescence intensity.


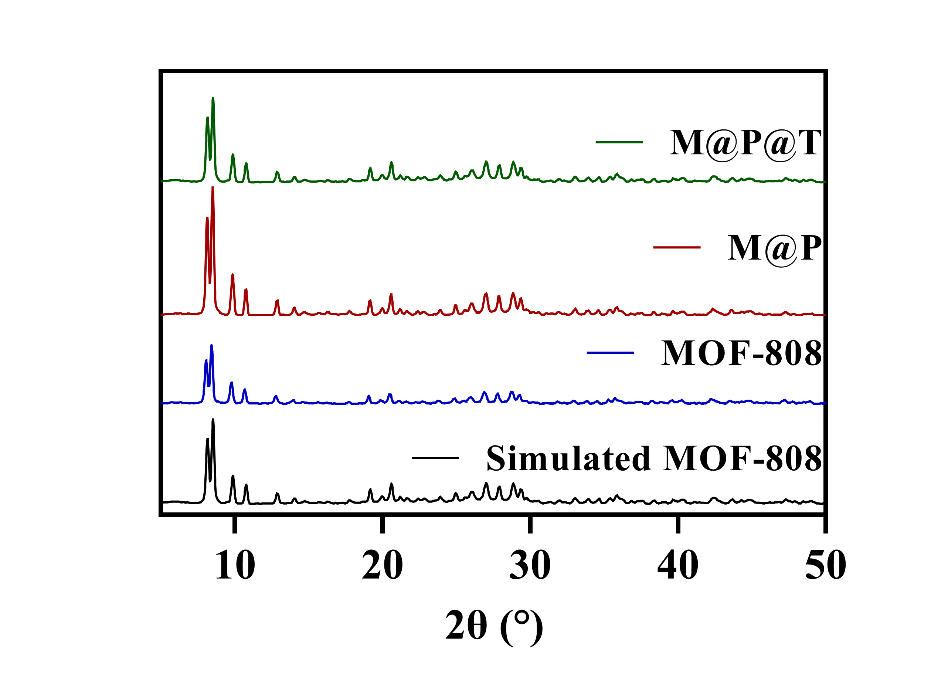


**Figure S2.** The PXRD profile of simulated MOF-808, MOF-808, M@P, and M@P@T.


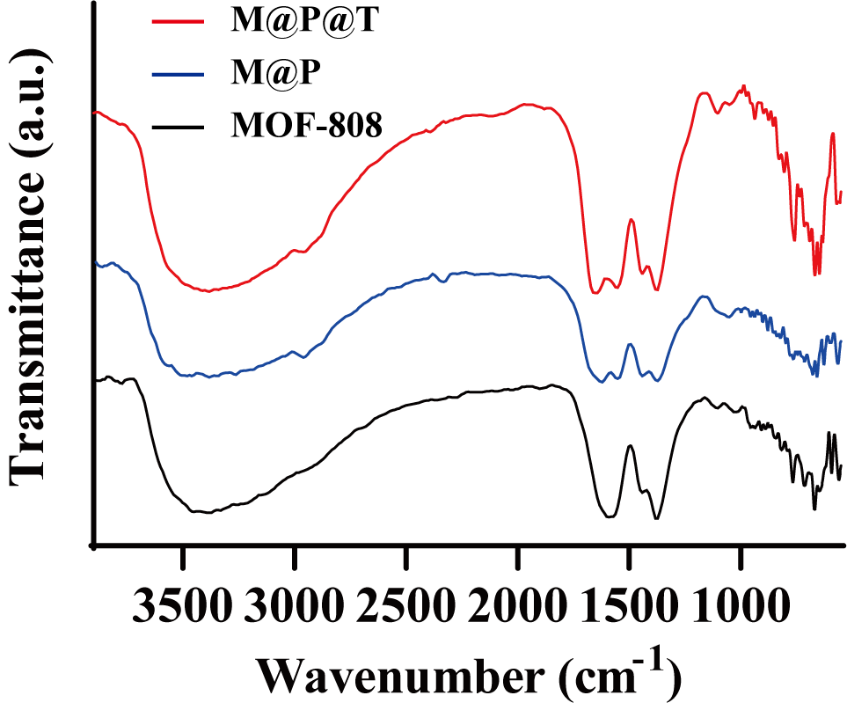


**Figure S3.** The FT-IR spectroscopy of MOF-808, M@P, and M@P@T.


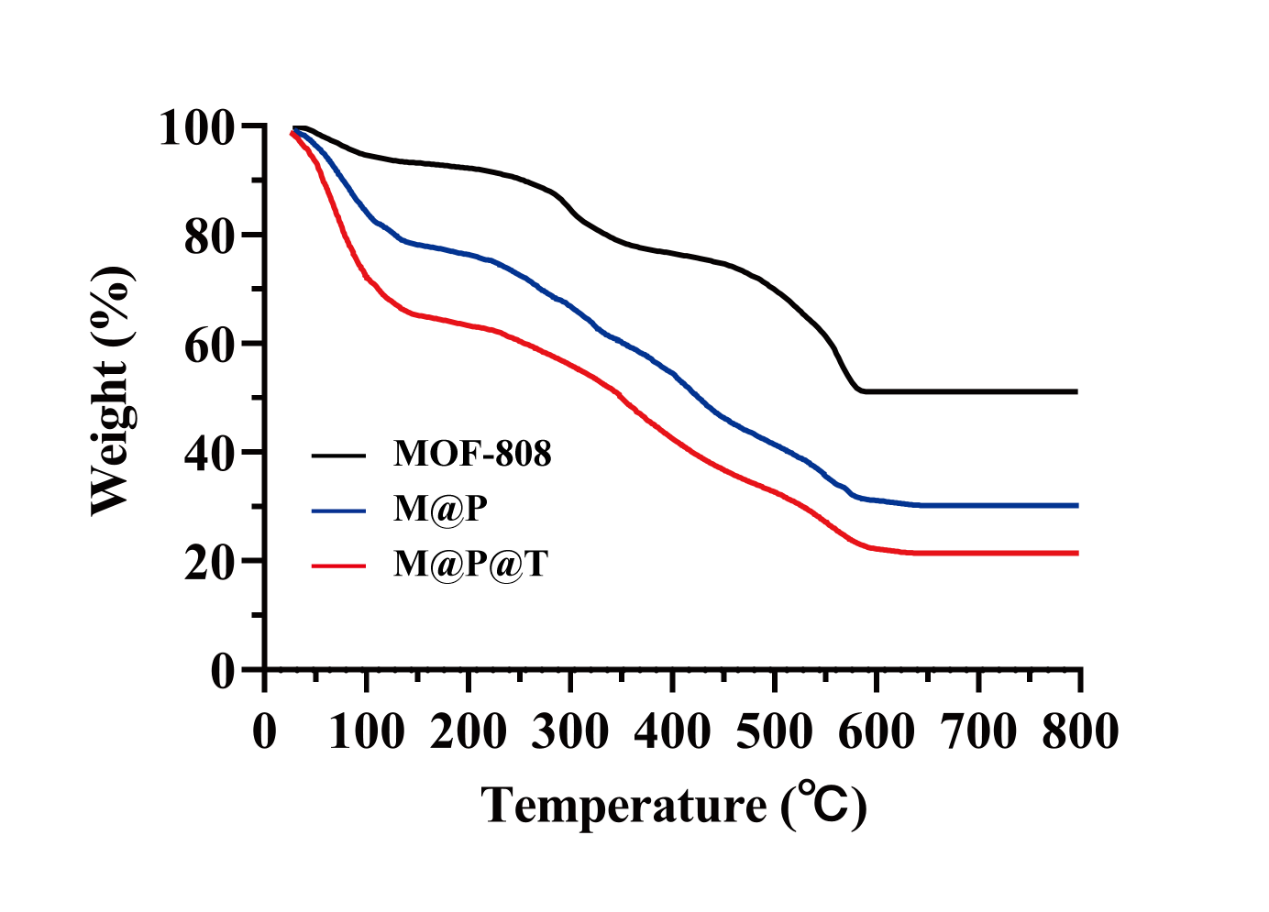


**Figure S4.** The thermogravimetric analysis (TGA) curves of MOF-808, M@P, and M@P@T.

**
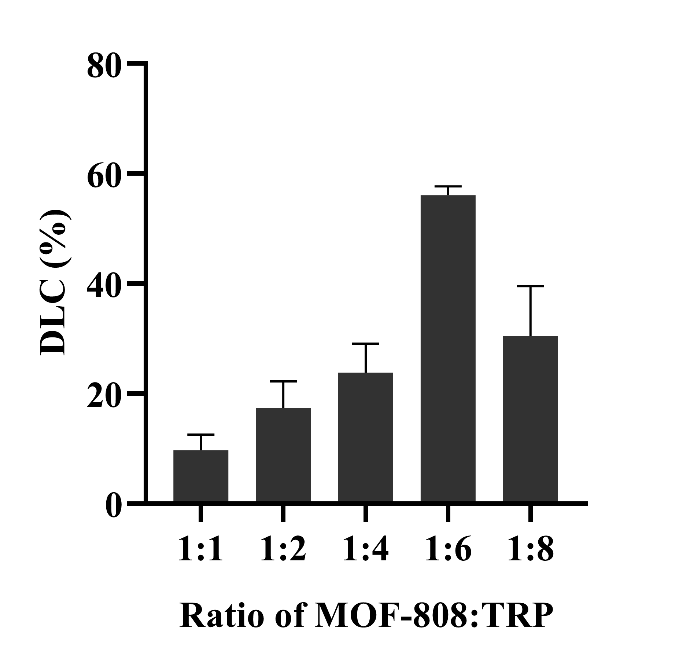
**

**Figure S5.** The changes of the drug loading content (DLC) of TRP as the ratio of MOF-808:TRP decreasing (n = 3 independent samples, mean ± SD).

**
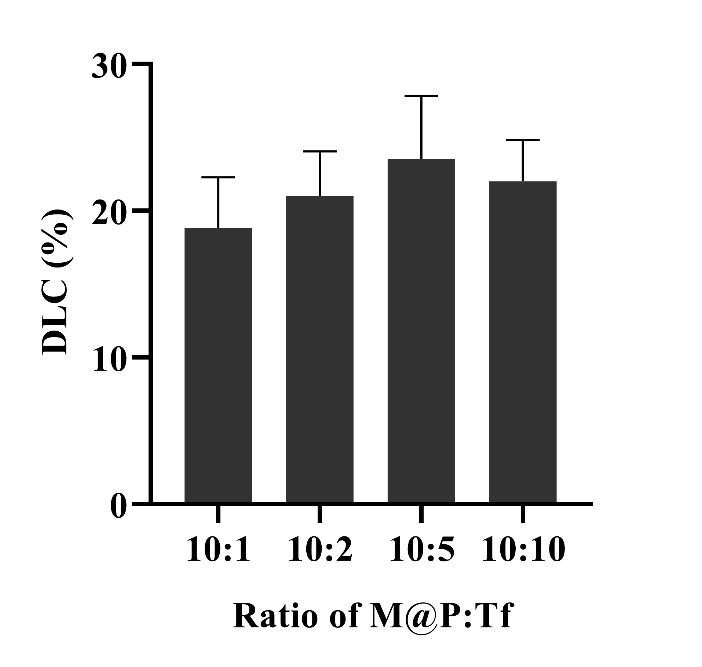
**

**Figure S6.** The changes of the DLC of Tf as the ratio of M@P:Tf increasing (n = 3 independent samples, mean ± SD).

**
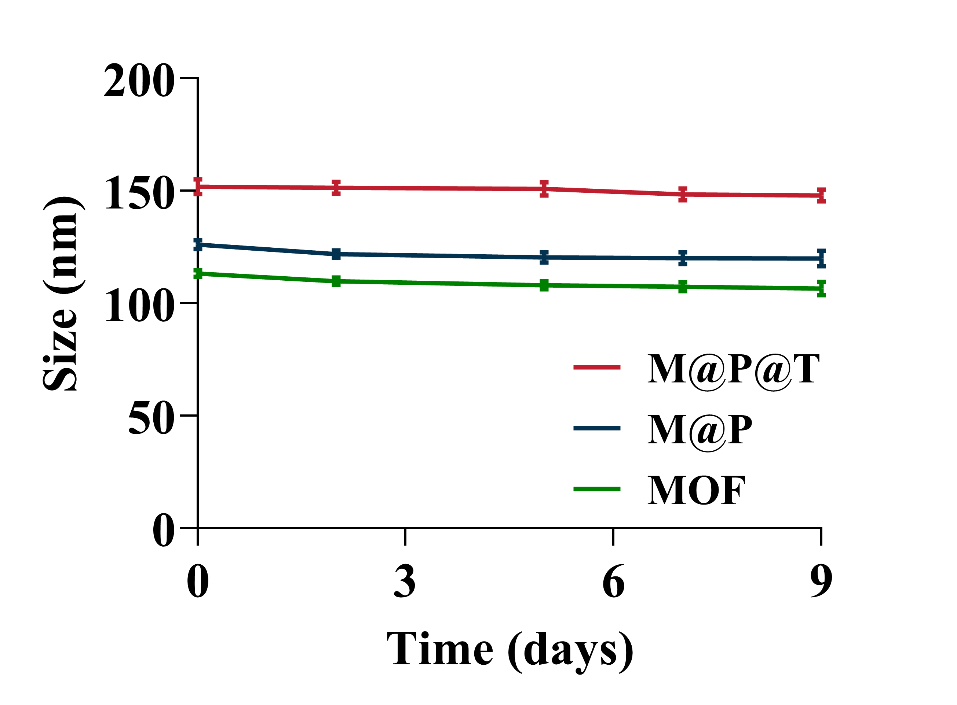
**

**Figure S7.** Size stability of the MOF-808-based nanosystems in ddH_2_O (pH = 2) after incubation at 37 °C for 9 days.


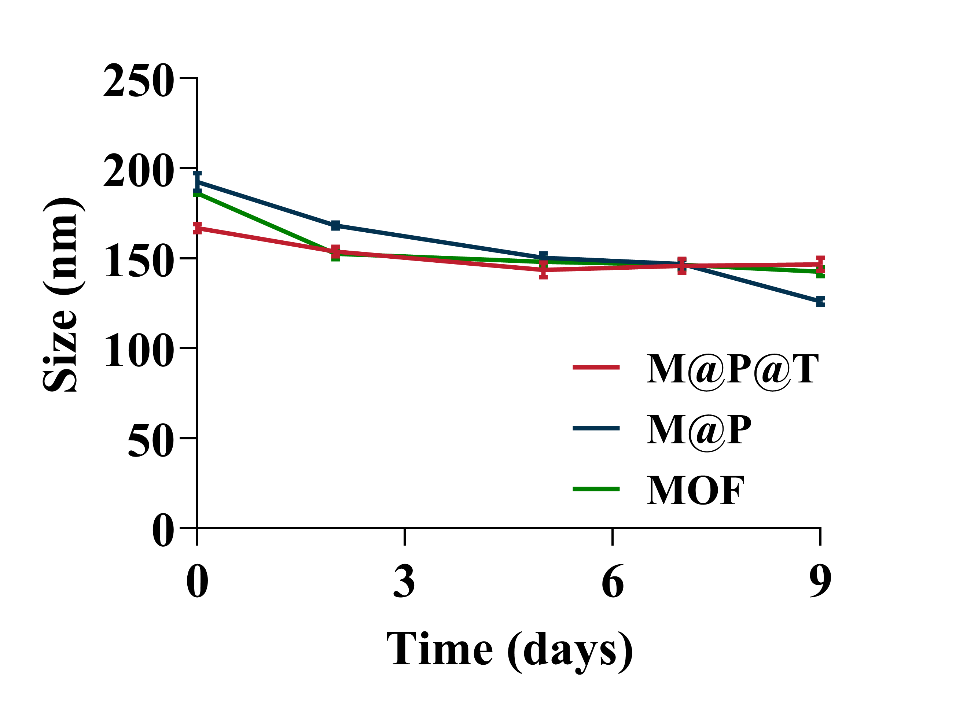


**Figure S8.** Size stability of the MOF-808-based nanosystems in DMEM (10% FBS) after incubation at 37 °C for 9 days.


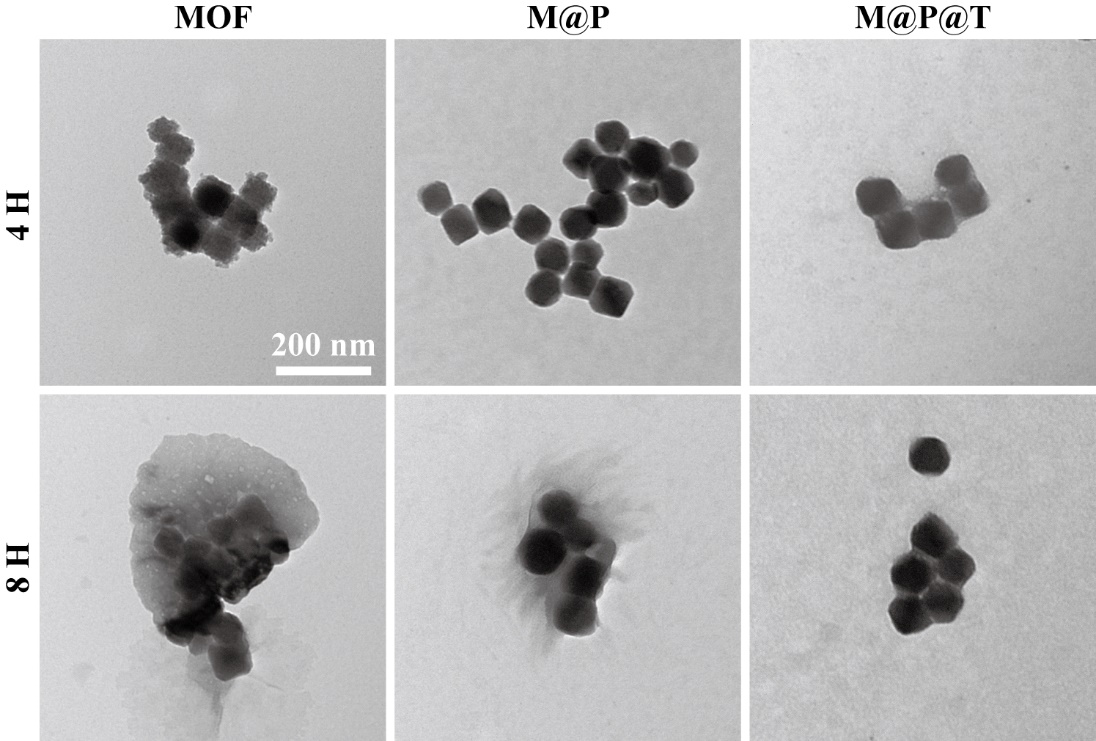


**Figure S9.** TEM images of MOF-808-based nanosystems after incubation in PBS for 4 and 8 hours. Scale bar, 200 nm.


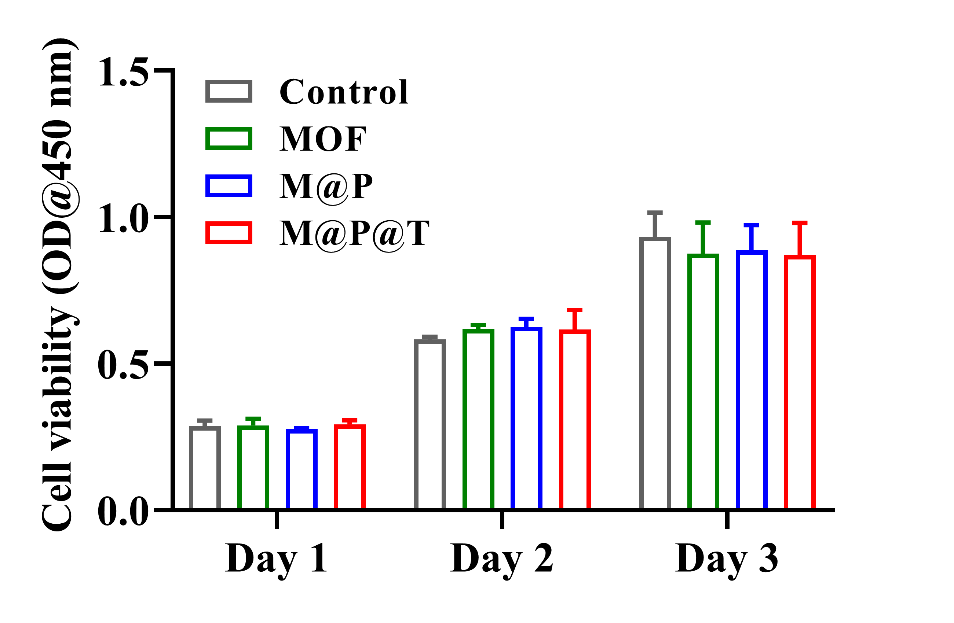


**Figure S10.** CCK-8 assay of MC3T3-E1 cells after treatment with different MOF-808-based nanosystems for 1, 2, and 3 days.


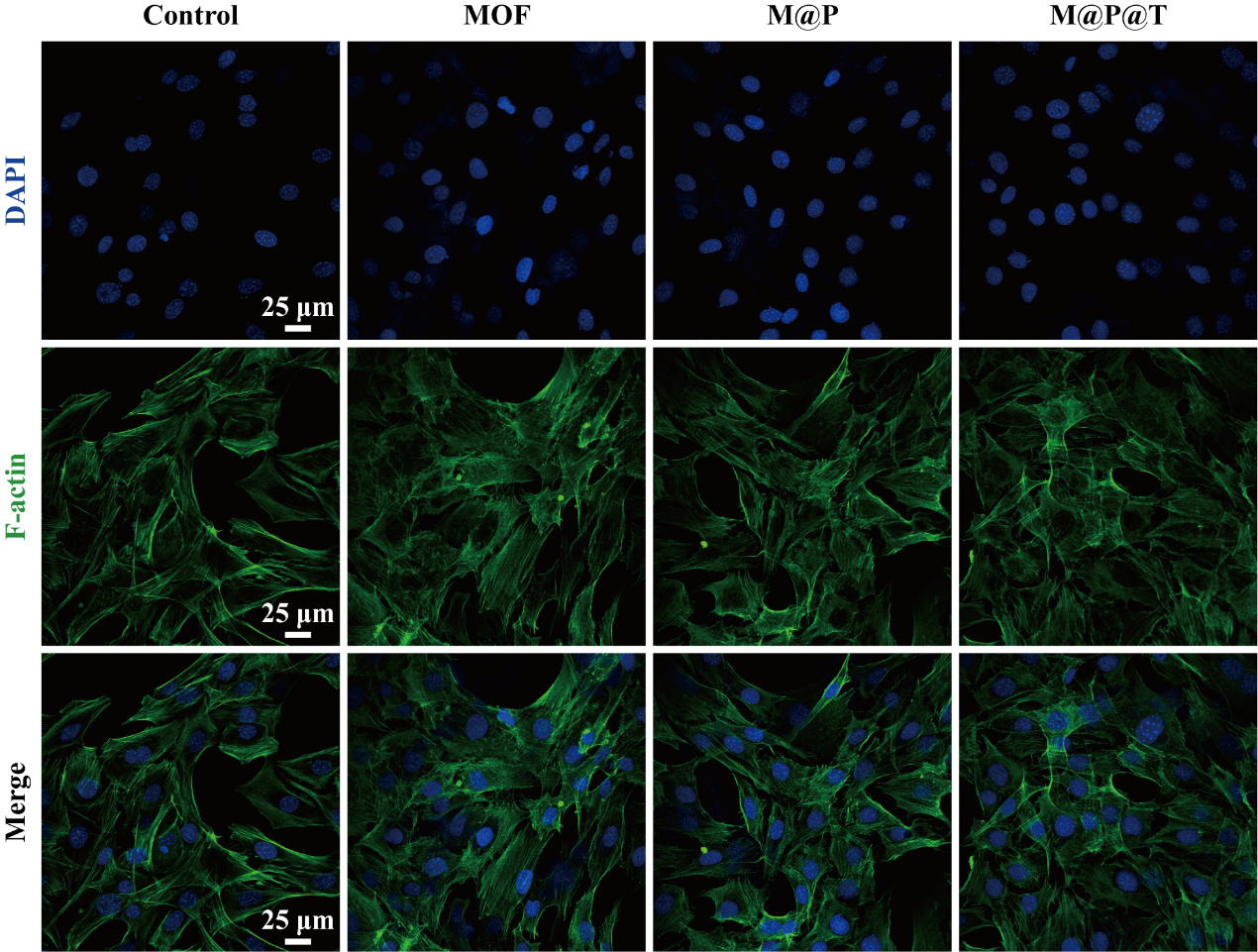


**Figure S11.** Nuclear (DAPI, blue) and cytoskeleton (F-actin, green) staining images after treatment with the MOF-808-based nanosystems. Scale bar, 25 μm.


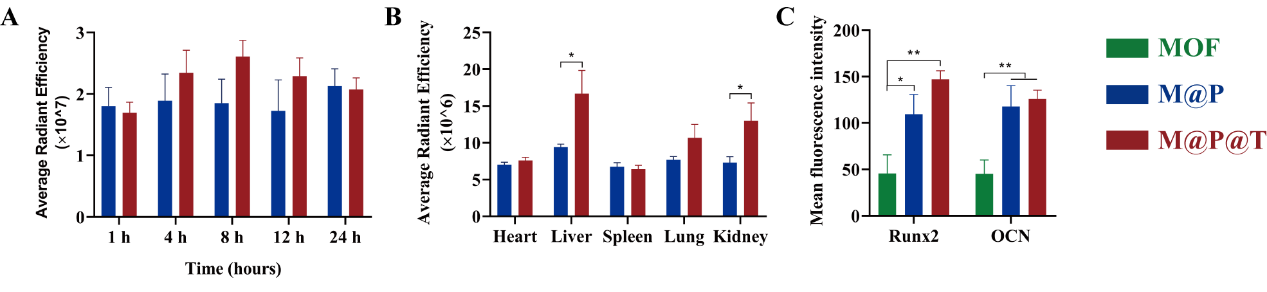


**Figure S12.** Semi-quantitative analysis of fluorescence intensity of intestine (A) and organs (B) using Living Image software. (C) Semi-quantitative analysis of immunofluorescence intensity using ImageJ software.

**
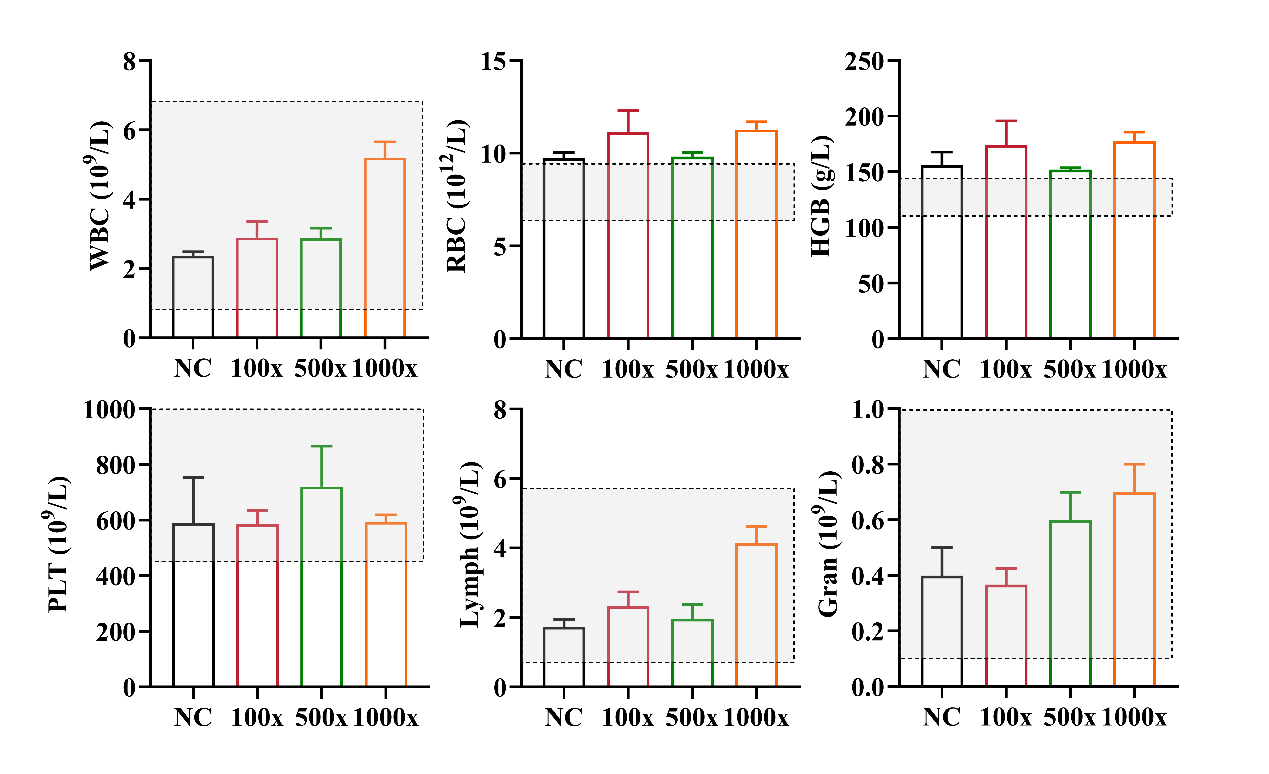
**

**Figure S13.** Routine blood analysis of mice after one-month oral administration with different doses of MOFs solution: NC (normal control group, administered with ddH_2_O), 100x (100 times therapeutic dose, 20 mg/kg MOFs solution), 500x (100 mg/kg MOFs solution) and 1000x (200 mg/kg MOFs solution), the gray dotted box is the normal range, n = 3.

**
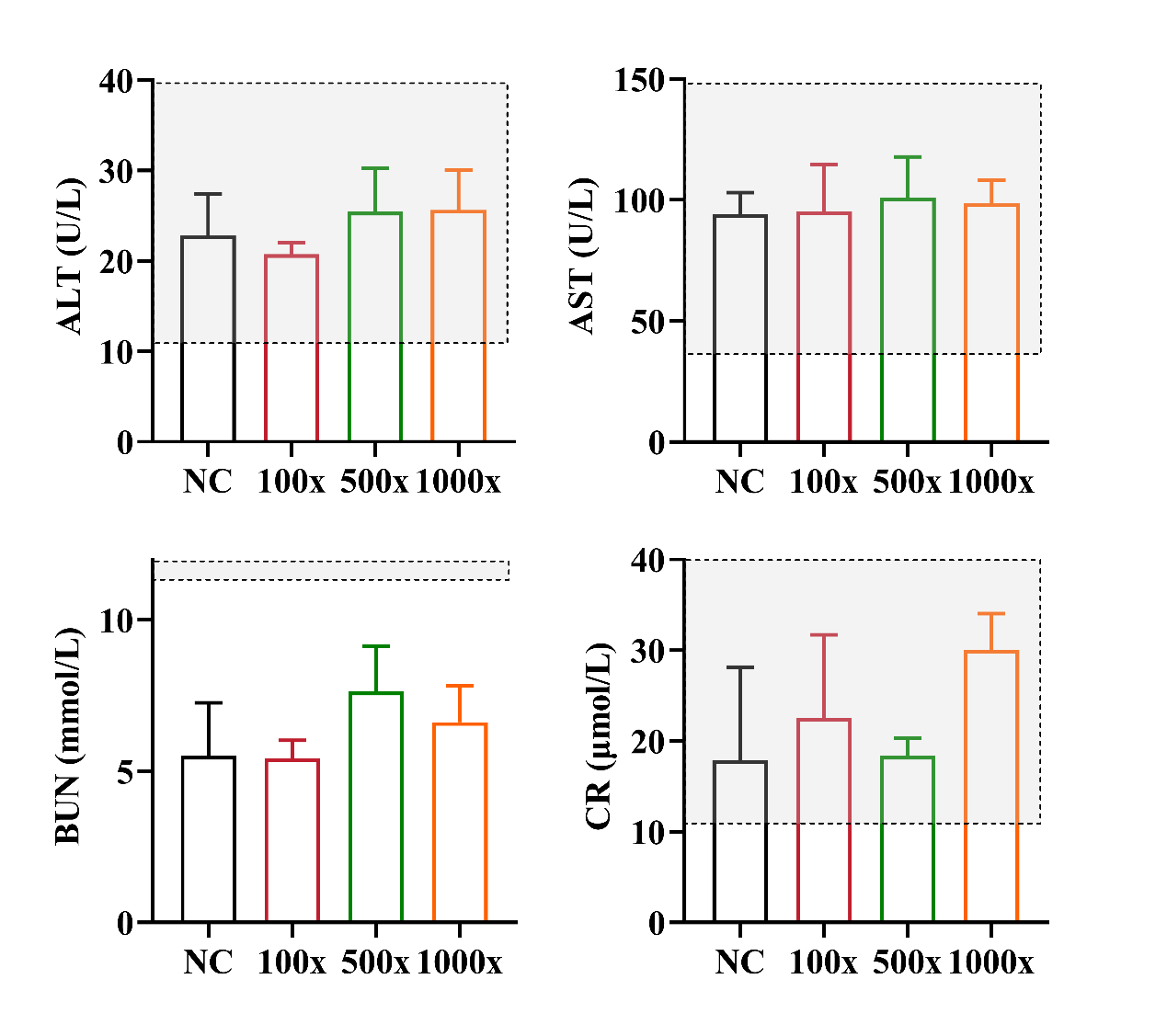
**

**Figure S14.** Liver and renal function analysis of mice after one-month oral administration with different doses of MOFs solution: NC (administered with ddH_2_O), 100x (20 mg/kg MOFs solution), 500x (100 mg/kg MOFs solution) and 1000x (200 mg/kg MOFs solution), n = 3.

**
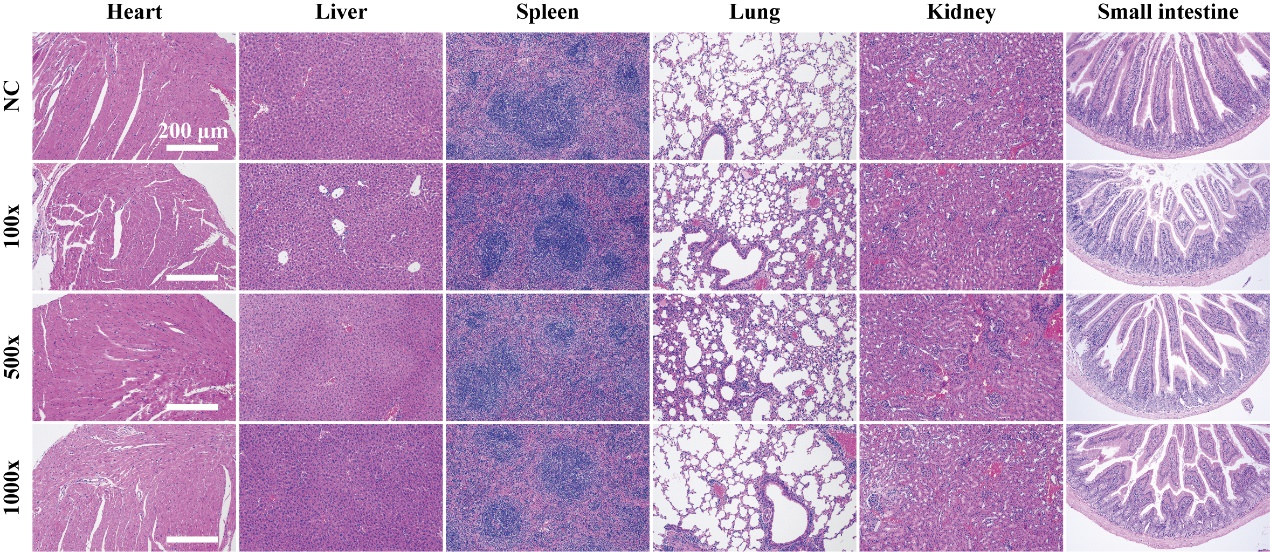
**

**Figure S15.** H&E staining images of the heart, liver, spleen, lung, kidney, and small intestine harvested after one-month of oral administration with different doses of MOFs solution: NC (administered with ddH_2_O), 100x (20 mg/kg MOFs solution), 500x (100 mg/kg MOFs solution) and 1000x (200 mg/kg MOFs solution). Scale bar, 200 μm.

**Table S2. Pharmacokinetic parameters of TRP in osteoporotic mice after subcutaneous injection of free TRP (20 μg/kg) and oral administration of M@P (200 μg/kg) and M@P@T (200 μg/kg) (n=5)**

| **Sample** | **Administration route** | **Dose (μg/kg)** | **AUC (pg*h/mL)** | **F (%)** |
| --- | --- | --- | --- | --- |
| TRP | s.c. | 20 | 117.1±11.2 | 100 |
| M@P | oral | 200 | 17.6±4.4 | 1.5 |
| M@P@T | oral | 200 | 240.1±22.5 | 20.5 |

**AUC: area under the plasma concentration−time curve; F%: relative bioavailability.**
